# Co-developing suicide prevention guidelines for pakistan: a mixed-methods Delphi consensus study

**DOI:** 10.1186/s12889-025-23942-3

**Published:** 2025-10-21

**Authors:** Tayyeba Kiran, Erminia Colucci, Samia Shahid, Sehrish Tofique, Suleman Shakoor, Zaina Imam, Nusrat Husain, Nasim Chaudhry

**Affiliations:** 1 Pakistan Institute of Living and Learning (PILL), Suite 201, 2nd Floor, Doctors Plaza, Khayaban-e-Iqbal, Block 9 Clifton, Karachi, Karachi City, 75600 Sindh Pakistan; 2 Middlesex University London, the Burroughs, London, NW4 4BT UK; 3 Division of Psychology and Mental Health, University of Manchester, Oxford Rd, Manchester, M13 9PL UK; 4 Global Centre for Research on Mental Health Inequalities Mersey Care NHS Foundation Trust, Kings Business Park, Trust Offices/V7 Buildings, Prescot, L34 1PJ UK

**Keywords:** Suicidal behaviours, Suicide prevention, Guidelines, Gatekeepers, LMICs, Asia

## Abstract

**Background:**

Suicide is a serious public health concern globally. Many suicide deaths occur in low- and-middle-income countries such as Pakistan, where the stigma related to mental health and suicidal behaviour is high, help-seeking is low, and availability of trained mental health professionals is limited. Community-based suicide prevention programmes such as suicide prevention first-aid guidelines are recognised as cost-effective approaches to strengthen the motivation of local partners within communities and lay public to act. However, there is no such evidence from Pakistan. Therefore, this study aimed to co-develop suicide prevention guidelines for gatekeepers to assist individuals in Pakistan experiencing suicidal ideation or behaviours. This will not only help to prevent or deter suicidal tendency among those experiencing suicidal thought/behaviours but also the stakeholders, especially mental health professionals.

**Methods:**

This Delphi expert consensus study was conducted in two phases: (i) development of a semi-structured questionnaire aimed to develop suicide prevention guidelines. This involved compilation of statements from existing guidelines developed for similar context, followed by a one-day multi-disciplinary stakeholder consultation to review and contextualise each statement. The questionnaire with final statement was translated into Urdu. (ii) Phase 2 involved the Delphi process to co-produce contextually relevant consensus-based set of suicide prevention guidelines endorsed by a diverse panel of experts including expert by profession and expert by experience. Statements describing suicide prevention guideline were rated by the participants in two Delphi rounds, using in-person and online approaches.

**Results:**

A total of 45 experts by profession and 27 lived experience experts from across Pakistan completed both rounds of Delphi. The initial compilation from existing guidelines led to a total of 460 statements, which increased to 564 statements after stakeholder consultation, to be rated in Round-1 of the Delphi. The total number of items describing guidelines accepted at Round-1 and 2 were 478. The statements are organised into eleven thematic sections, including the identification of suicide risk and its severity, initial support for individuals at risk, communication strategies for engaging with suicidal individuals, safety planning, and confidentiality protocols. Stakeholders recommended the inclusion of context-specific guidelines, such as recognising culturally relevant warning signs (e.g., verbal or behavioral expressions of feeling unloved or being forced into an unwanted marriage), advising first responders to adopt a friendly and non-judgmental tone, and assessing the urgency of intervention based on the individual’s mental health status.

**Conclusion:**

The guidelines developed as result of this mixed-method research has successfully engaged stakeholder to contextualise guidelines for Pakistan such as by adding culturally appropriate examples of warning signs, methods used for self-harm and suicide, and reasons of self-harm etc. and Delphi survey to reach consensus. These guidelines co-adapted through consultations with experts by profession and experience will inform much needed public health initiatives to increase awareness and education and build capacity in a wide range of stakeholders across sectors for suicide prevention in Pakistan.

**Supplementary Information:**

The online version contains supplementary material available at 10.1186/s12889-025-23942-3.

## Introduction

Suicide is a serious global public health issue, with more than 700,000 suicide deaths reported globally in 2019 [1]. Majority of these suicide deaths (more than 77%) are in low and middle-income countries (LMICs) where more than 70% of global population lives [1]. More than 39% of all suicides globally occur in Southeast-Asia where 8.5% of world’s population lives [2]. The health services related economic costs due to suicidal behaviours are enormous including the costs of medical emergency treatment, general and psychiatric hospitalisations, outpatient care, informal care costs, and productivity losses [3].

Official data on suicide is limited in Pakistan [4]. Although the estimated suicide rate in Pakistan is low (4.4/100, 000 individuals) [5], recent data indicates that suicide is a major public health concern in Pakistan [6]. Suicidal behaviours and suicide were considered illegal acts until very recently, when the Senate abolished the provision of punishment for those who attempt suicide [7]. Nevertheless, suicidal behaviours are socially and religiously condemned in Pakistan and social stigma in form of family’s fear of negative impact of such behaviours on family’s honour is reported as a major barrier to help-seeking [8] in addition to other barriers such as lack of awareness, financial constraints, and a low literacy rate [9]. Lack of awareness about the role of psychological services further exacerbate the problem [8]. A recent evidence from Pakistan highlights that majority of the adults who self-harm did not communicate this to anyone prior to self-harm [10].

There is little research on prevention of self-harm and suicide in Pakistan [11, 12]. Only 0.4% of the national health budget is allocated to mental health. Service level challenges have also been reported including limited access to psychological services and lack of training arrangements for health professionals such as general practitioners and emergency care staff [8, 13]. There are around 400 qualified psychiatrists in Pakistan, mostly concentrated in urban cities [14] leading to a geographical disparity as 64% of Pakistan’s population resides in rural areas [15]. In addition, the country has only 5 major psychiatric hospitals, 650 inpatient mental health units, and 3800 outpatient clinics [16]. People also resort to alternate sources, such as traditional, spiritual and faith-based healers in the absence of specialised mental health services [17] or also in addition to it there are successful examples of partnership between healers and healthcare workers in LMICs [18]. Moreover, Pakistan is home to a large number of internally displaced people (IDPs) and refugees, which pose an additional challenge to an already stretched healthcare system [19]. This vulnerable population in LMICs is at heightened risk of suicide [20]. Therefore, the WHO LIVE LIFE implementation framework has emphasized the importance of population-level awareness and educational campaigns [21].

Suicide prevention is not only important for individuals and families but also benefits the well-being of society, the health care system, and the economy at large [22]. Ensuring healthy lives and promoting well-being for all ages is the third goal of the United Nations Sustainable Development Goals (SDGs) [23]. The suicide rate is an indicator for target 3.4 of the SDGs i.e., reduce premature mortality from non-communicable diseases by one third by 2030 through prevention, treatment and the promotion of mental health and well-being [23]. To achieve this goal, the WHO (2021) has recommended a public health approach to identify and provide treatment to high-risk individuals.

Community-based suicide prevention programmes are recognised as cost-effective approaches which can strengthen the motivation of participating regional partners within communities to take action [24]. In order to improve community capability to reduce suicide, gatekeeper training is one of the most widely used strategies [25] and it was included, for instance, as one of the selective interventions in the milestone report ‘Suicide: A global imperative’ by WHO (2014). The latest LIVE LIFE framework also recommended role of community gatekeepers in early identification of suicide risk, assessment, management and follow-up [21]. Gatekeepers are community members who are in regular contact with people at risk of suicide and can open the “gate” to support services [26]. In context of Pakistan, community gatekeepers including community leaders, Lady Health Workers, teachers, and religious scholars who played role in other health projects may play vital role in suicide prevention. Gatekeepers training involves training people who are not necessarily clinicians to be able to identify people experiencing suicidality and refer them to appropriate services [25] or support them till the crisis has passed (e.g. when appropriate services are not available) [27]. Key aspect of gatekeeper training is to improve knowledge, skills, and attitudes of trainees to improve their intentions to intervene with someone at risk of suicide [28].

Recognising the need to tailor gatekeepers resources and training to the cultural contexts (Colucci, in press), suicide prevention first aid guidelines have been developed to help gatekeepers and lay public to identify and provide support to high-risk groups in different countries such as Philippines [29], Japan [30], Sri Lanka [31], China [32], Indonesia [33], Brazil [34] and also for specific groups such as for immigrants or individuals with refugee background [35]. The existing suicide prevention first-aid guidelines include information for first aiders on general and context specific suicide warning signs, identification of suicide risk and its seriousness, guidance for first aiders on how to talk to suicidal individuals to offer initial assistance, safety planning, confidentiality, practical guidance for suicidal individuals on passing time during crisis, adolescents specific and gender specific guidelines [30, 31, 35]. Evidence supports that training of suicide prevention first aid guidelines lead to raised awareness and improve participants’ knowledge on assisting suicidal individuals, in addition to promoting positive attitude towards suicide prevention [27].

Given the differences between countries in language, culture, healthcare systems and available resources for mental health in general and suicide prevention in particular, the suitability of suicide prevention guidelines developed for other countries for use in Pakistan is currently unknown [36] and cultural adaptation is warranted [37]. Therefore, this Delphi consensus study was conducted to co-develop the suicide prevention guidelines to help gatekeepers and lay public to identify those who are at risk such as those experiencing suicidal thoughts or displaying suicidal behaviours and offer support. The Delphi consensus method, a widely used method in mental health research, provides a systematic way to enable recommendations and decisions to be made by incorporating practice-based evidence with evidence-based practice [38].

## Methods

### Design

Delphi expert consensus method [38] was used to elicit consensus on statements to be included in the final suicide prevention guidelines for Pakistan. The study was conducted in following two phases:

### Phase 1: development of questionnaire

#### Compilation of statements from existing guidelines

We compiled a bank of statements from the guidelines already developed in various countries within the region, particularly those developed for Muslim contexts such as Indonesia [33]. The statements in existing guidelines were developed through a robust process that included extensive literature search, focus group discussions with relevant stakeholders and one-to-one interviews with lived experience experts. Existing mental health first aid guidelines for suicide prevention from other countries were also reviewed, i.e., Sri Lanka [31], Philippines [29], Indonesia [33] and guidelines for immigrants and people with refugee background [35].

The compilation led to a total of 460 statements in 6 sections: [1] identification of suicide risk [2], assessing seriousness of the suicide risk [3], initial assistance to suicidal people [4], talking to a suicidal person [5], specific to adolescents and [6] gender specific. Statements in these sections indicate warning signs, potential first aid actions (e.g. what the first aider should do or should not do) or statements that suggest what a first aider should know (e.g. if a person is not suicidal, asking them cannot put the idea of suicide in their head).

#### Stakeholder consultation meeting

One-day stakeholder consultation meeting was held in 2018 that was led by EC who had previously led the development of suicide prevention first aid guidelines for India [39], Japan [40], Philippines [41], Sri Lanka [42], Indonesia [33] and guidelines for immigrant and people from refugee backgrounds [35]. This stakeholder consultation meeting was attended by 24 multidisciplinary stakeholders: the mental health professionals (psychiatrists and psychologists with representation from ethnic minorities) both from UK and Pakistan, community engagement expert, social workers, teachers, and a nurse from Pakistan. Inclusion of non-mental health experts at this stage was a deliberate decision aligned with both public health principles and the realities of suicide prevention in low-resource settings like Pakistan. Suicide prevention often depends not only on clinical intervention but also on early identification, support, and referral—roles that are frequently played by community members, teachers, religious leaders, and frontline healthcare providers who are not specialised in mental health. Furthermore, the co-adaptation process aimed to ensure cultural relevance, community ownership, and feasibility of implementation. Non-mental health stakeholders bring critical insights into local beliefs, stigma, help-seeking patterns, and barriers to care. These stakeholders are part of suicide prevention division of Pakistan Institute of Living and Learning, already engaged in suicide prevention work [12, 43], and invited through e-mail to participate in stakeholder consultation. The contact was maintained through e-mails and telephone.

We refined the initial questionnaire through following step:

1. Review of each statement in small groups of 5–6 members to analyse cultural relevance and shortlisting of statements for each category in the guidelines i.e., identification of immediate suicide risk, assessing urgency of risk, initial assistance, talking with the suicidal person, ensuring safety, passing time during the crisis, what the first aider should know, confidentiality, adolescent-specific and gender-specific statements.
2. Each statement was then discussed in a large group with all the attendees and each group shared feedback on modifications required, which were audio-recorded. All group members also provided paper copies of the guidelines with their notes to the research fellow (TK).
3. Two members (TK and EC) then finalised a questionnaire incorporating suggestions provided by the stakeholders. This led to a total of 564 statements and two additional items to ask participants to add any comments they have (The file is attached as “Supplementary file - SFAG - First Round”). The revised set of statements were categorised into 11 sections, consistent to guidelines for Indonesia: (1) identification of suicide risk, (2) assessing seriousness of suicide, (3) initial assistance to a suicidal person, (4) talking to a suicidal person, (5) safety planning with suicidal people, (6) ensuring safety for suicidal people, (7) passing time during a crisis, (8) what the first aider should know in providing suicide first aid to a suicidal person in Pakistan, (9) confidentiality, (10) section specific to adolescents and, (11) gender specific section.
4. The final questionnaire was then translated into Urdu. The Urdu translation of the questionnaire followed standard procedure of translation (47).

### Phase 2: Delphi process

#### Selection of the expert panel

For Delphi study, it is important to choose a group of people who have relevant expertise related to the research questions. The experts should have knowledge, experience and relevance to the issue related to research question [45]. A group size of 40 to 45 is recommended [38]. For this study we followed the procedure used for the guidelines for people from immigrant and refugee backgrounds [35] and recruited two groups of experts: experts by profession and lived experience experts. The group of experts by profession included Pakistan-based mental health professionals, front-line health workers who encounter persons contemplating suicide, general physicians, teachers, social workers, community health workers, religious scholars, legal personnel working with self-harm and suicide related cases. The lived experience experts group included individuals with personal experience of self-harm and carers. Experts could belong to both categories. The inclusion of a diverse panel of experts is considered a key indicator of methodological rigor in Delphi studies [46].

### Delphi rounds

Potential experts by profession were invited by the research team across Pakistan (Lahore, Rawalpindi, Quetta, Peshawar, Karachi, Hyderabad) through already established partnership developed during mental health research including suicide prevention research [12, 43]. Snowball technique was also used to identify and approach some experts such as religious scholars, teachers etc.

For lived experience experts, participants were identified and approached from previous suicide prevention projects [12, 43] and through our network of community engagement officers.

An initial meeting was held between the researchers (master level psychologists) and the potential participants, either face-to-face or by telephone, depending on experts’ convenience. During the meeting, researchers provided an overview of the study including its purpose, procedures involved and asked for their preference for a printed copy of the questionnaire, or a personalised link hosted by the Survey Monkey website. Informed consent was obtained from all the participants either by signing a paper copy or through an electronic version. Assent was obtained from those less than 18 years of age.

Participants were also offered the option to complete the survey during a face-to-face meeting with research team either at a research office or at participant’s home. Most of the lived experience experts completed the survey either in one or two face-to-face meetings with the research team whereas all the experts by profession completed the survey online in their own time.

The survey had a one-month completion deadline from the date of the invitation and reminders were sent for those who had nor accepted/rejected or had started but not completed the survey.

For those who completed the questionnaire through printed copy, their responses were entered into the Survey Monkey platform by the research team. All participants of Delphi survey were reimbursed for their time. Certificate of participation was also sent to the participants through email (experts by profession) and in a face-to-face meeting (expert by experience).

During the Round-1 survey, participants received a questionnaire that included two sections. The first section aimed to collect basic socio-demographic characteristics of the participants. In the second section, they were asked to rate each statement using the response categories: essential, important, don’t know, unimportant, or should not be included. At the end of each section, there was also a comment box where participants were invited to add comments on the existing items and suggest any additional item that was not indicated in the existing list but was particularly relevant to the Pakistani context. The consensus threshold was adopted from the previous Delphi study [35], i.e., the statements that were rated as ‘essential’ or ‘important’ by 80% or more of the members of both the professional and lived-experience groups of participants; or by 70–79% of one group and 80% or more of the other were included in the guidelines. Statements rated as ‘essential’ or ‘important’ at least by 70% of both groups but not by 80% or more of either were re-rated in the Round-2 questionnaire. If a statement was rated as ‘essential’ or ‘important’ by less than 70% of the members of either group, it was excluded.

During Round-2, a questionnaire comprising of the statements that were included for re-rating from Round-1 and new items that were generated by the participants from the comments and suggestions in Round-1 were sent to participants. Participants in Round-2 were the same as those who participated in Round-1, with a 100% retention rate. At the end of this round, any item that reached the 80% consensus criterion in both groups, or 70–79% in one group but 80% or more in another group, was selected for inclusion in the guidelines. The new items reaching between 70 and 79% of consensus by the members of one or both groups (but was not rated 80% or more by the other group) were supposed to be used to create the Round-3 questionnaire. However, ratings in Round-2 either met the criteria for the statement to be included in the guidelines or were rejected. Therefore, Round-3 was not required.

### Ethical considerations

Ethics approval of the study, which included informed consent and assent processes and a distress policy, was obtained from the Pakistan National Bio-ethics Committee (NBC) (Ref: No.4–87/NBC-513/20/324).

## Results

A total of 114 experts by profession were approached by the research team and 45 completed the survey. The response rate is similar to what was reported in the Delphi survey in Indonesia. Of these 45 experts by profession, two were community engagement officers with experience in suicide prevention research, two Lady Health Workers, three teachers who previously participated in a suicide prevention trial in Pakistan as a gatekeeper, three social workers, two emergency rescue officers, five psychiatrists, nine clinical psychologists, six nurses, three General Physicians, one police personnel, one lawyer, six religious scholars, and two occupational therapists. All these professionals have a background in suicide prevention work in Pakistan. Those who did not participate in the survey, either did not respond to the research team (*n* = 15), refused to participate due to busy schedule (*n* = 43), or initially agreed to participate but did not complete the survey despite reminders (*n* = 11).

A total of 36 lived experience experts were invited to participate by the research team. Of these, 27 completed the survey: 18 people with history of suicidal behaviours and 9 family members (5 family members of attempt survivors and 4 bereaved family members). See Table 1 for the demographic characteristics of the participants. None of the participants indicated to belong to both experts' panels.

**Table 1:** Demographic characteristics of the experts by profession and experience

	Experts by Profession	Experts by experience
Age, Mean (SD)		
Gender		
Male	21 (46.7%)	11 (40.7%)
Female	24 (53.3%)	16 (59.3%)
Age group		
16 to 30	08 (17.8%)	18 (69.2%)
31 to 45	27 (60.0%)	05 (19.2%)
Above 45 years	10 (22.2%)	03 (11.5%)

Round-1 survey started with 564 statements. At the end of this round, a total of 472 statements were included in the guidelines, 73 statements were excluded (e.g., warning sign - unusual engagement with suicide related material such as movies, games, news, etc., dramatic change of habits.), 19 were to be re-rated (e.g., warning sign - Sudden or dramatic increase in depressed/sad mood (including crying more than usual or lack of smiling) and 15 new statements were suggested by the participants (See Table 2).

**Table 2:** New statements suggested by the participants in Round-1 survey

1	An important warning sign for suicide is if a person is expressing in words or actions that they are a failure in life.
2	An important warning sign is if a person makes wills or starts distribution of property between family members.
3	An important warning sign for suicide is if a person is expressing in words or actions that they are feeling sad or depressed.
4	An important warning sign for suicide is if a person is expressing in words or actions an overwhelming level of mental distress and/or excessive worry (or worries).
5	An important warning sign for suicide is if a person says he/she is upset but don’t know the reason
6	An important warning sign for suicide is if a person is expressing in words or actions that they feel hated (e.g. by other people in general or someone specifically).
7	An important warning sign for suicide is if a person expresses resentment or other negative feelings about having married someone not of their own choice.
8	An important warning sign is if they are no longer taking care of cleanliness of their room or home or their personal hygiene.
9	The first aider should ask the person about any drastic change in their social interactions such as meeting friends etc
10	The first aider should talk with the person in a friendly tone.
11	The first aider should not talk about religion (e.g. to try to make them feel guilty) but focus on other reasons to stay alive (e.g. parents and children).
12	The first aider should determine the urgency of taking action (e.g. emergency or mental health referral) based on knowledge about the person’s mental health disorder (e.g. psychotic or bipolar).
13	If the person is addicted to alcohol and drugs they should not remove what is their daily dose and look for professional help for their addiction.
14	The first aider should not suggest any activity and location that could potentially trigger suicidal person's upsetting thoughts.
15	More likelihood of denial (including of feeling suicidal) among males.

Round-2 survey was comprised of 34 statements including items to be re-rated and new items. At the end of this round, a total of 25 statements were included in the guidelines and 9 were excluded. As indicated above, there was no item to be re-rated in Round-3 and no further suggestions were given by the participants (See Table 3 for examples of items accepted for inclusion in the guidelines).

**Table 3:** Examples of items accepted for inclusion in the guidelines

Statements	Round
Section 1. IDENTIFICATION OF SUICIDE RISK	
Threatening to hurt or kill themselves.	1
Describing themselves as a burden to others or expressing feelings of guilt or shame (e.g. stating that others will be better off without them).	1
Expression of feeling trapped, like there is no way out.	1
Sudden or dramatic increase in depressed/sad mood (including crying more than usual or lack of smiling).	2
Giving away valued possessions and getting affairs in order, including asking others to take on responsibility for the care of people or pets.	2
Section 2. ASSESSING SERIOUSNESS OF THE SUICIDE RISK	
The first aider should determine the urgency of taking action based on recognition of suicide warning signs.	1
The first aider should not let the suicidal person convince them that it is not serious or that they can handle it on their own.	1
The first aider should ask the suicidal person if they have a plan for suicide.	1
The first aider should be aware that there are certain groups of people who are more at risk for suicide such as elderly people who are chronically ill and living alone.	1
The first aider should ask the suicidal person if there have been changes in their employment, social life, or family.	1
Section 3. INITIAL ASSISTANCE TO A SUICIDAL PERSON	
The first aider should not put themselves in any danger while offering support to the suicidal person.	1
The first aider should take the suicidal person to the nearest safe place (e.g. hospital).	1
The first aider should help the suicidal person understand that they have control over their suicidal thoughts.	1
Section 4. TALKING TO A SUICIDAL PERSON	
The first aider should tell the suicidal person they care and want to help.	1
The first aider should give the suicidal person their undivided attention.	1
Encourage the suicidal person to do most of the talking.	1
The first aider needs to allow the suicidal person to talk about their reasons for wanting to die.	1
Section 5. SAFETY PLANNING WITH SUICIDAL PEOPLE	
The first aider should develop a safety plan with the suicidal person.	1
The safety plan should be clear, outlining what will be done, who will be doing it, and when it will be carried out.	1
Section 6. ENSURING SAFETY FOR SUICIDAL PEOPLE	
The first aider must gain the person’s trust before removing the means of suicide.	1
If the first aider can’t get the suicidal person to agree to hand over the means of suicide (e.g. pills, gun, razor), they should try to take these things secretly.	1
Section 7. PASSING TIME DURING A CRISIS AMONG SUICIDAL PEOPLE IN PAKISTAN	
Ask the suicidal person to postpone the decision to suicide.	1
Encourage the suicidal person to spend time with their significant others (e.g. family, friends, or religious leaders).	1
The first aider should ask if the suicidal person is involved in spiritual and/or religious practices (e.g. pray, Wird/Zikr, Ijtema, etc.) and encourage these practices to pass time.	1
Section 8. WHAT THE FIRST AIDER SHOULD KNOW IN PROVIDING SUICIDE FIRST AID TO A SUICIDAL PERSON IN PAKISTAN	
Of how commonly suicide occurs.	1
Of the link between suicide and mental illness.	1
Section 9: CONFIDENTIALITY AMONG SUICIDAL PEOPLE IN PAKISTAN	
The first aider must never agree to keep the suicidal person’s suicidal plans a secret.	1
The first aider should not keep the person’s suicidal thoughts a secret from potential helpers but should discuss with the person whether other details should be confidential.	1
Section 10. SPECIFIC TO ADOLESCENTS	
The first aider should not leave an adolescent who is feeling suicidal on their own.	1
Discuss with the suicidal person what actions they should take to get help.	1
In order to reduce suicide risk, it is important for the first aider to try to solve the suicidal adolescent’s problems.	2
The first aider should not try to take on the suicidal adolescent’s responsibilities.	2
Section 11. GENDER SPECIFIC	
The first aider should be aware of different risk factors for a suicidal woman such as domestic violence, postnatal depression, and interpersonal conflicts.	1
The first aider should be aware of different risk factors among a male suicidal person such as alcohol misuse and substance abuse, financial difficulties.	1

The total items accepted at Round-1 and 2 were *N* = 478. The accepted items were compiled by the research team in a narrative format to create the Suicide Prevention Guidelines for Pakistan, available in both English and Urdu versions (attached as supplementary files).

## Discussion

The increasing burden of self-harm and suicide in LMICs warrants the development of cost-effective and easily scalable solutions for prevention of suicide in these settings [27]. This mixed-method research has shown that the suicide prevention guidelines for Pakistan are similar to those developed for other Muslim countries such as Indonesia [33]. However, stakeholders during phase 1 and participants of phase 2 suggested changes in terms of contextually specific examples such as for warning signs, passing time during crisis, emergency contact details (based on difference in healthcare systems) etc.

Statements related to warning signs—such as “talking or writing about death, dying, or suicide (including making unexpected jokes about these topics, or leaving a suicidal note on social media platforms such as WhatsApp or Instagram, or in the form of a poem or letter)” and “unusual engagement with suicide-related content such as movies, games, or news”—which are present in international guidelines [33] but were rejected by participants in Round 1 of the Delphi survey in Pakistan, are consistent with existing evidence from Pakistan indicating that over 65% of individuals presenting with self-harm report no history of either direct or indirect communication about self-harm or suicidal ideation [10]. Moreover, like guidelines for Indonesia, in the section “Passing Time During the Crisis,” participants in this study rejected the statement suggesting the use of sleeping pills by a suicidal person—for example, “encourage the suicidal person to take some sleeping pills, as they should be feeling better by the time they wake up.” This rejection is supported by existing evidence indicating the widespread use—and often misuse—of psychoactive substances, particularly benzodiazepines, in Pakistan, commonly as a form of self-medication [47]. The participants’ response may reflect a concern about reinforcing or legitimising such practices, given the associated risks and high prevalence.

Religious context of Pakistan is reflected through stakeholders’ endorsement of statements related to role of religion. The recommendation that “the first aider should not talk about religion” highlights importance of avoiding religious arguments intended to induce guilt or shame—for example, reminding the person that suicide is forbidden in Islam or that they will be punished in the afterlife. Such messages can increase distress and hinder help-seeking. In contrast, statements encouraging the use of religious practices, such as prayer, were endorsed as a form of voluntary coping, but only when initiated or welcomed by the suicidal person. This distinction reflects the difference between imposing religious beliefs and supporting culturally meaningful coping mechanisms that the person themselves finds comforting. Previous evidence also highlights the role of following Islamic rituals in reducing distress and depression [48].

In the section specific to suicidal adolescents in Pakistan, participants rejected statements such as “If the suicidal adolescent says that the situation is not serious or that they can handle it on their own, the first aider should respect this,” and “If the suicidal adolescent wants to be left alone, and can assure the first aider of their safety, the first aider should agree.” This rejection may be explained by existing evidence indicating that suicidal behaviours in this population are typically solitary in nature [49] and most often occur within or near the home environment [50]. These circumstances may present critical opportunities for timely intervention, and participants may have perceived such guidance as potentially increasing the risk of harm by delaying necessary support.

These culturally appropriate suicide prevention guidelines serve as an opportunity to promote the WHO recommended ‘task-shifting’ approach in LMICs to tackle the mental health treatment gap i.e., training non-specialists such as nurses, teachers, and community health workers (CHWs) (i.e., local gatekeepers) to provide mental health services under the guidance of specialist [51]. A systematic review including 21 studies exploring the views and experiences of service users and healthcare providers concluded that task-sharing in LMICs (specifically in Africa and South-Asia) was largely considered acceptable and feasible [52]. A comprehensive literature review proposed that identification of gatekeepers and their training can potentially influence four factors that are important for suicide prevention: knowledge, perceptions about suicide prevention, reluctance, and self-efficacy [53]. Capacity building of gatekeepers to identify individuals at risk of suicide and to mobilise support has been greatly emphasised [21]. However, the gatekeeping programmes must be tailored to the target population [54].

Considering the increasing rates of suicide in Pakistan, limited availability of trained mental health professionals and established role of gatekeeping programmes in the world, this Delphi study makes an important contribution towards suicide prevention programme by developing culturally and locally relevant suicide prevention guidelines. The participation of multidisciplinary experts by profession and lived experience experts adds strength to the cultural relevance and local buy-in. By using culturally sensitive language and involving non-mental health stakeholders in the Delphi panel, the guidelines promote socially acceptable ways to talk about suicide and mental distress. Moreover, the guidelines are designed to be implemented by a wide range of gatekeepers—including community health workers, teachers, and religious leaders who are often the first point of contact in underserved or remote areas. The retention of participants throughout the Delphi process is an indication of the engagement of stakeholders as well as their commitment towards suicide prevention in Pakistan.

The way forward following development of these Suicide Prevention Guidelines for Pakistan involves developing dissemination plan in collaboration with key stakeholders including Department of Education, Ministry of Health, academic institutes, religious organisations (including Madrassa), non-profit organisations to raise awareness about the guidelines and develop local capacity and capability throughout the country through gatekeeper trainings. A comprehensive training programme must be developed that should include a Train-the-Trainer Toolkit to be implemented nationally (Colucci, in press). The training programme will be developed using the processes described for suicide prevention first aid guideline gatekeepers training in Syria [27]. Context specific training material will be developed to include guidelines booklet, infographics and training videos to help training participants to identify warning signs. For evaluation, all training activities will include pre-post assessments. In addition to this, plans for monitoring and evaluation will include pilot implementation in selected communities, development of fidelity checklists, mixed-method evaluation plans that will include qualitative feedback from attendees, strengthening collaboration and partnerships with healthcare system to track referral patterns and suicide related outcomes. Furthermore, awareness campaigns should be co-designed using these guidelines for the public on how to identify key warning signs and suicide first aid actions (to dos and not to dos) taking into account the unique need of vulnerable groups such as ethnic minorities experiencing specific risk factors (forced displacement and discrimination). Future implementation of these guidelines will also consider inclusion of gatekeepers from diverse ethnic backgrounds. This may require community consultation and co-design when adapting materials for specific minority groups and other vulnerable groups such as LGBTQ+, particularly those with historical marginalisation or limited access to mental health services. Moreover, it is important to note that these guidelines are intended as an immediate, practical response tool to assist individuals in crisis and do not aim to replace the need for upstream policy reform or broader public health.

The modified Delphi process used in this study, as well as that used in Indonesia, showed the value in consulting with key stakeholders and people with lived experience of suicide to modify and add context-specific items in the questionnaire before starting the rating process through the Delphi rounds. Furthermore, in spite of some differences, most of the statements in the Pakistan guidelines are similar to those developed in Indonesia [33]. It could therefore be viable, if similar finding were repeated, to extrapolate a core set of warning signs and key actions to be used as basis for guidelines gatekeepers training for other predominantly Muslim countries (Colucci, in press).

## Conclusion

As indicated by the WHO (2014) and shown in research in Pakistan [8, 10, 49, 55], there are several reasons and pathways that lead to self-harm and suicide and, therefore, suicide prevention must be multi-sectorial and multi-component. Increasing public awareness and education and building capacity in a wide range of stakeholders including the gatekeepers across sectors (from mental health to gender and domestic violence-related to welfare services, schools and primary care) using these contextually relevant suicide prevention guidelines is an essential and urgent priority in suicide prevention in Pakistan.

## Supplementary Information

Supplementary Material 1

Supplementary Material 2

Supplementary Material 3

## Data Availability

The current version of the guidelines and a table containing the endorsement rate of each item are available as supplementary files.

## Abbreviations

LMICs: Low- and Middle-Income Countries
IDPs: Internally Displace People
UN: United Nations
SDGs: Sustainable Development Goals
WHO: World Health Organisation
CHWs: Community Health Workers
