# Supplementary material for: Co-developing suicide prevention guidelines for pakistan: a mixed-methods Delphi consensus study
Source: BMC Public Health. 2025 Oct 21;25:3536. doi: 10.1186/s12889-025-23942-3 (PMC12538931; doi:10.1186/s12889-025-23942-3)
Supplement: Supplementary file 1 — Supplementary Material 1 [file 12889_2025_23942_MOESM1_ESM.pdf]

Suicide Prevention Guidelines for Pakistan

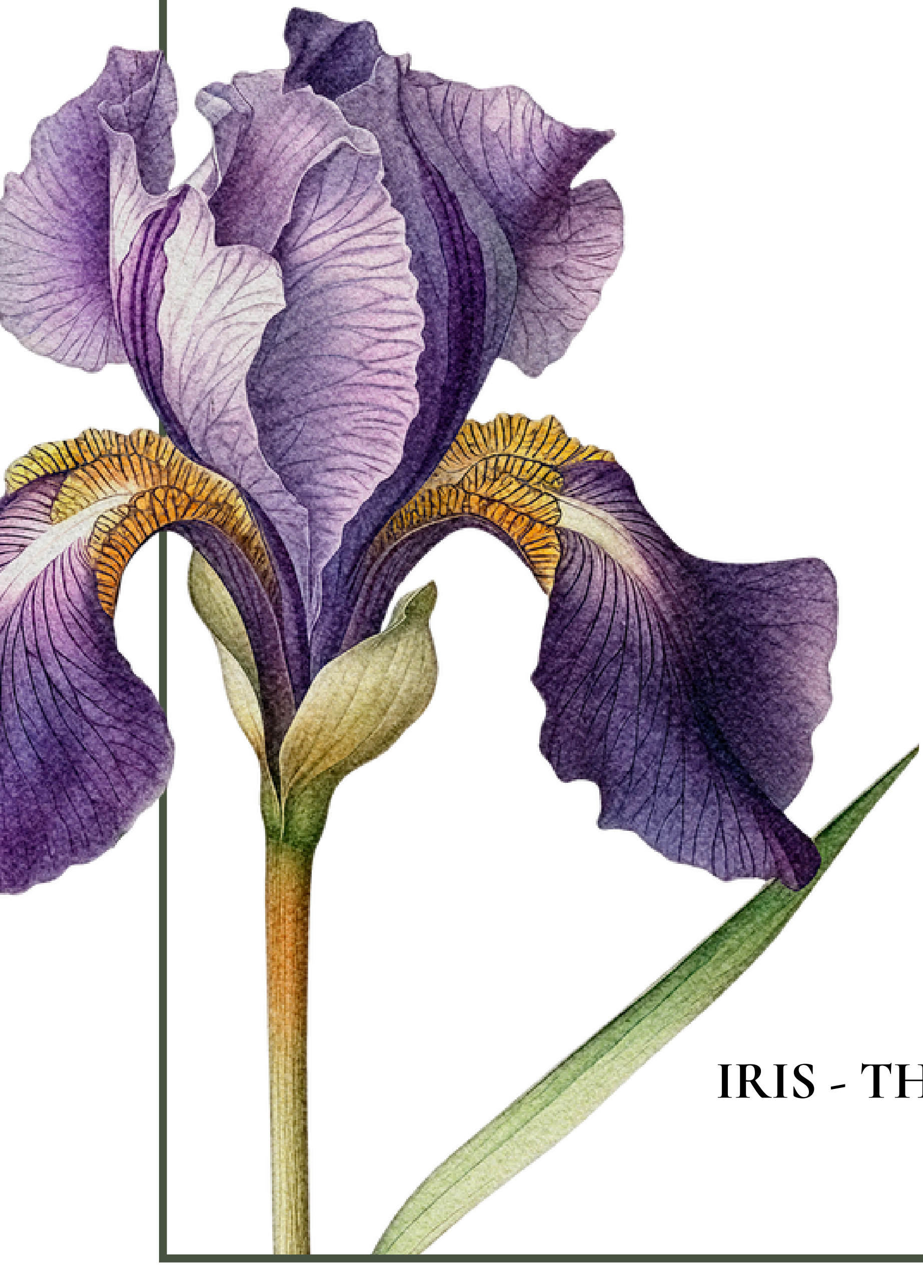

IRIS - THE FLOWER OF HOPE

Pakistan Institute of Living and Learning
Health Department Government of Sindh
Middlesex University London
NCRSP National Centre for Research on Suicide Prevention
MANCHESTER 1824 The University of Manchester

SUICIDE PREVENTION GUIDELINES FOR PAKISTAN

Preventing self-harm and suicide in Pakistan is possible. Often, those considering suicide are seeking relief from their suffering rather than truly wanting to end their lives. Engaging in a simple, supportive conversation with someone who is suicidal can have a profound impact and might save a life. It is essential to recognise your potential to help in such situations, as your actions can make a life-saving difference.

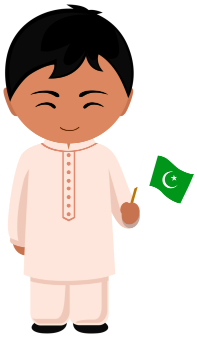

ROSE - THE FLOWER OF LOVE

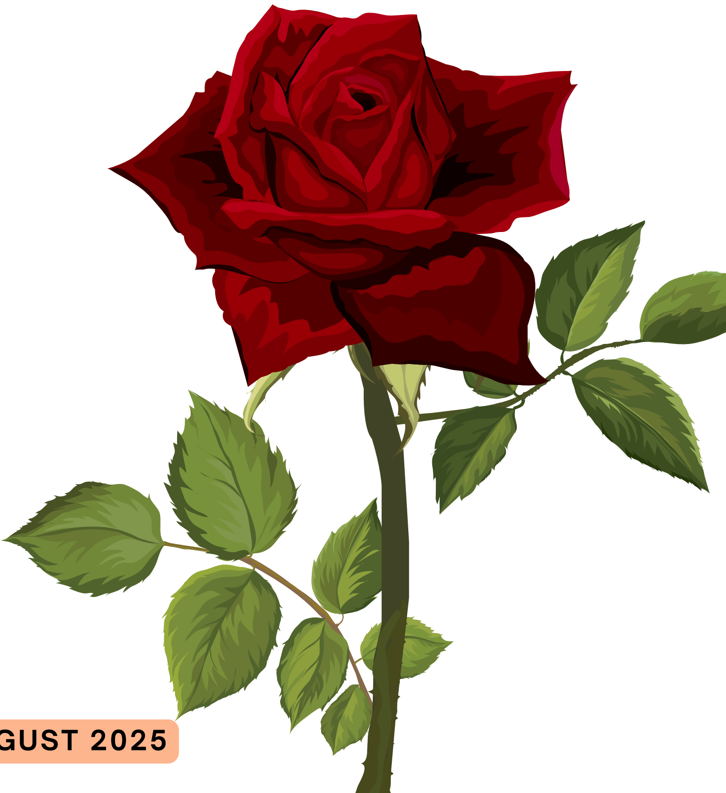

VERSION 2 - 4TH AUGUST 2025

HOW CAN I RECOGNISE IF SOMEONE IS EXPERIENCING SUICIDAL THOUGHTS?

It is important to learn about thoughts, feelings and behaviours that may be a sign that someone is thinking about suicide. These feelings and behaviours are called "warning signs". These warning signs are the earliest *detectable indication of increased risk for suicide in the near future (i.e. within minutes, hours or days).* It is crucial for you to understand and recognise the warning signs of suicide.

NOTE: If the person has already acted on their thoughts of suicide, this should be treated as a medical emergency, you should administer first aid if qualified to do so, and call emergency services, asking for an ambulance.

SUICIDE WARNING SIGNS

A PERSON MAY SHOW A BIG CHANGE IN MOOD, BEHAVIOURS AND/OR APPEARANCE, SUCH AS:

EXPRESSING IN WORDS OR ACTIONS

- Hopelessness or feeling worthless or that their life is worthless
- A sense of guilt or self-blame for something that has happened, or belief of being a burden to others (e.g. saying "Others will be better off without me")
- Feeling trapped, like there is no way out
- Telling significant others that they want to end their life
- Lack of reasons to live or having no purpose in life
- Lack of interest in or plans for the future
- Having the desire or hope that they will die (including praying that God may take their life)
- Believing suicide to be the only solution to their problems or being unable to find an alternative solution to a problem.
- Feelings of extreme dislike or hatred of oneself
- Strong sense of feeling alone and/or cut off from family and friends (feeling lonely or isolated)
- Sudden or dramatic increase in depressed/sad mood
- Wanting to disappear
- Believing their 'time has come' and/or that it is time to rest
- Being convinced that they are a failure in life

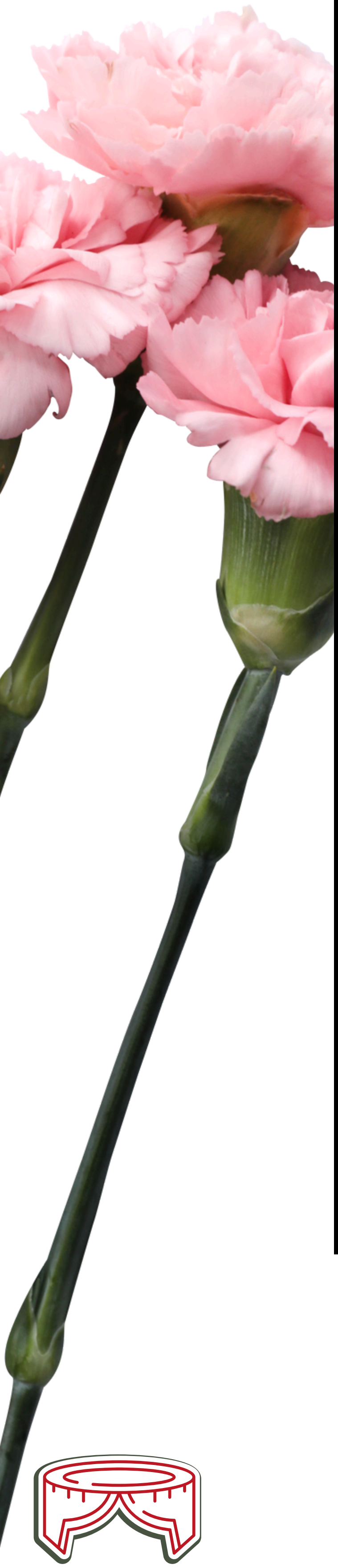

- An overwhelming level of mental distress and/or excessive worry (or worries)
- Feeling hated (e.g. by other people in general or someone specifically)
- Resentment or other negative feelings about having married someone not of their own choice.
- Isolating themselves including wanting to sit and spend time in the dark, not wanting the lights on.
- Becoming more sensitive, easily angered, irritated
- Refusing to eat for days
- Stop expressing themselves (such as not sharing any feeling with others).

A person may threaten to hurt or kill themselves, for example:

- Looking for a way to kill themselves (e.g. seeking access to pills or poisons, weapons or other means), including asking information about possible suicide methods (e.g. "would 100 mg of this kill me?" or “would I die if I jumped from that building?").

A PERSON MAY BEHAVE IN WAYS THAT ARE LIFE-THREATENING OR DANGEROUS, FOR EXAMPLE:

01 Acting recklessly or engaging in risky activities, seemingly without thinking

02 Engaging in self-injurious behaviours such as cutting, burning themselves, poisoning [e.g. drinking cleaning agent (such as bleach, phenyl) or mosquito repellent (such as mospel), or insecticide (such as wheat or rat pill etc.)] or hitting their head against a wall

03 Daring someone to kill them (e.g. “just shoot me”)

04 Putting oneself in a situation with high risk of being killed (e.g. confronting armed officers, like the police).

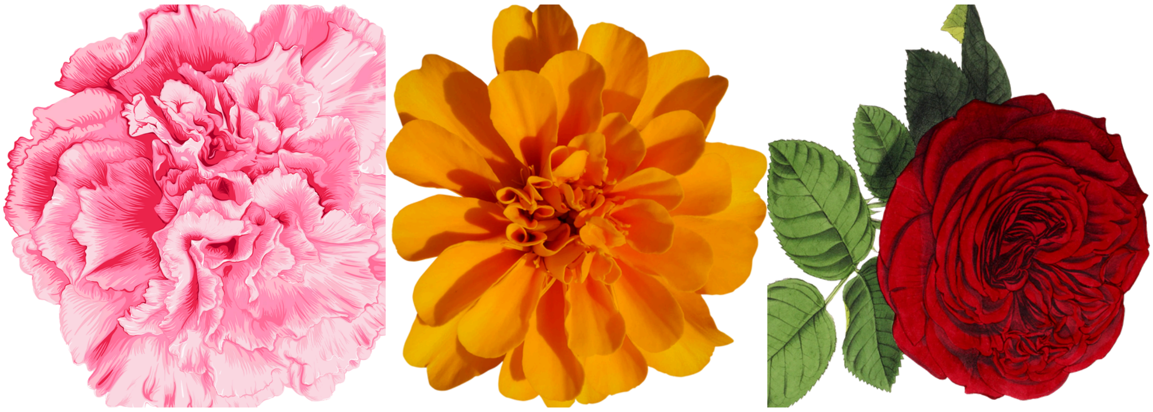

A PERSON MAY TRY TO SET THEIR AFFAIRS AND RELATIONSHIPS IN ORDER:

01 Giving away valued possessions, getting affairs in order, including asking others to take on responsibility for people or pets

02 Making wills or start distribution of property between family members

03 Saying goodbyes to friends and family.

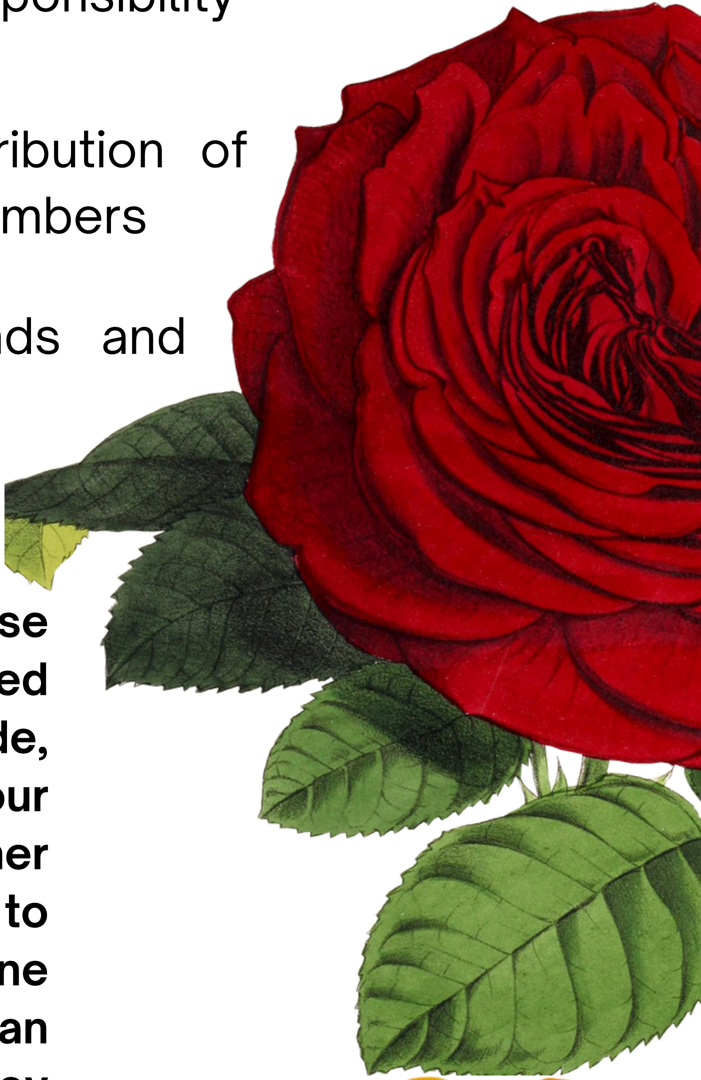

If you have noticed some of these warning signs and you are concerned a person may be at risk of suicide, you need to talk to them about your concerns. If you are not sure whether what you have noticed is a reason to be alarmed, you could ask someone who knows the person better than you, if they are worried too or if they know the person's problem.

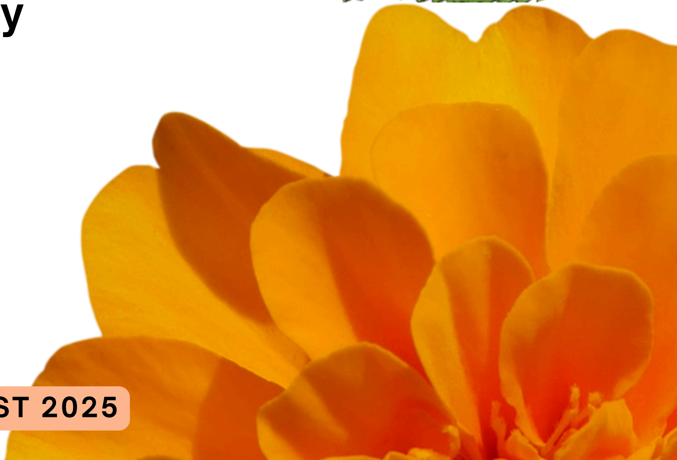

GETTING READY TO APPROACH THE PERSON

Be aware that suicide is common but preventable. Thoughts of suicide are temporary, and anyone could have these thoughts. It is important for you to understand reasons why people have thoughts about suicide as, for most people, suicidal behaviour is a request for help. They do not want to die, they simply do not want to live with pain, believing they have no choice but to die by suicide. They think suicide is a better choice than being a burden to others. Be mindful of the fact that these people are less likely to seek help directly (try to understand the reason why they do not ask for help, such reasons may include stigma) but do point out the warning signs to their family and close friends if necessary. This means that a first step for you is to recognise the warning signs of suicide, and a second step is ensuring access to suicide crisis resources, including the local services that can assist in response to people at risk of suicide (i.e. hospitals, mental health clinics, mobile outreach crisis teams, suicide prevention helplines, or local emergency services).

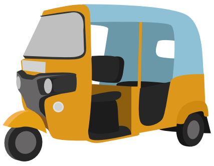

Your physical presence is not compulsory, you can also help through the phone but do not assume that the person will seek help on their own. Choosing a private place to talk to the person and a slot when there is sufficient time to discuss their concerns is important. However, in case of immediate risk of suicide, it is advisable not to wait to find sufficient time to talk to the person. This delay in intervening may keep the person at risk. In all crisis situations it is important to act promptly, try to talk to other people who know the person, to see if they also have concerns.It is important to avoid the assumption that the person will get better without help.

Be mindful of your own attitudes about suicide and the impact of these upon your ability to help, e.g. beliefs that suicide is wrong, forbidden in Islam and/or other religion, or if it is a rational option. Similarly, if you are concerned about someone who is from a different religion and/or cultural background to your own, you should get to know a little about their religious/cultural beliefs and attitudes towards suicide before approaching the person, as there are differences in cultural beliefs and attitudes about suicide, such as in Pakistani religious and legal aspects, although suicide has been decriminalised in Pakistan since December 2022.

Be aware, even if you are ready to approach the person, you are not responsible for the actions or behaviour of someone else and cannot control what they might decide to do.

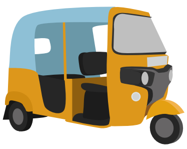

MAKING THE APPROACH

Explain the person the behaviours/warning signs that are the reason of your concern for them. However, it is important to avoid raising the topic of suicide with the person during an argument or if they are really upset because this may end up getting a bad reaction and distancing them. As widespread stigma is attached to self-harm and suicide, using terms to describe suicide that promote stigmatising attitudes must be avoided, for instance, 'commit suicide' or refer to a suicide attempt as having 'failed' or been 'unsuccessful' or using judgmental words like ‘crazy’. Therefore, using appropriate language when referring to suicide by using the terms 'suicide' or 'died by suicide' is important.

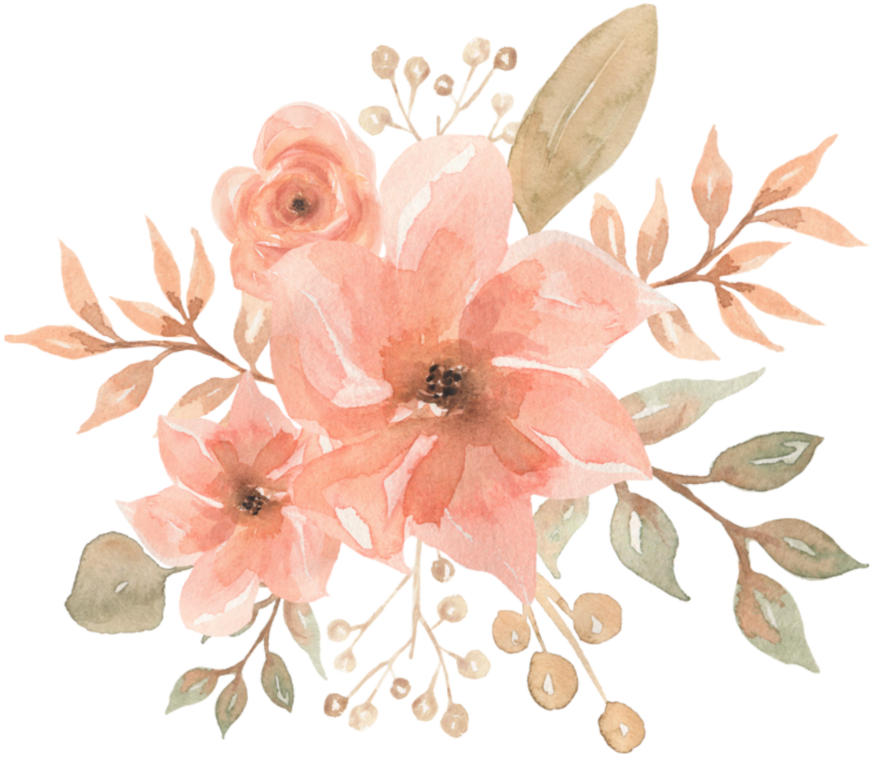

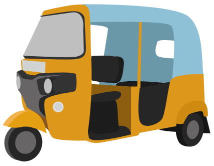

ASKING ABOUT SUICIDAL THOUGHTS

Do not underestimate your abilities to save a life by helping a person and the best way of helping is talking openly with them about suicidal thoughts and feelings. Rest assured, talking about suicide will not 'put the idea' into someone's head and it will not increase the risk that they will act on these thoughts. Understand the importance of “asking” as, unless someone tells you, the only way to know if a person is thinking of suicide is to ask. Asking them about suicidal thoughts will allow them the chance to talk about their problems and show them that somebody cares. Even if you have a mild suspicion that the person is having suicidal thoughts, ask them. It is important to ask the person directly about thoughts of suicide, even if you feel uncomfortable in doing so. Moreover, in case you notice any wounds and you are unsure whether the noticed wounds are from an injury or from a suicide attempt, you should ask the person directly about the wounds.

Do not let the fear of saying the wrong words or of not saying the perfect words stop you from talking to the person. While talking to the person, you should not be concerned about the exact wording, however, be aware that the stigma associated with suicide might refrain them from disclosing their suicidal thoughts and seeking help. Hence, you should begin the conversation by asking the person about how they are feeling and allow the person time to discuss their negative feelings before asking about suicidal thoughts. Ask that person indirectly at first:

"Do you ever wish you would not wake up in the morning?".

If the person says "yes", ask them directly (i.e. are you having thoughts of suicide?) without dread or expressing negative judgement. You should respect the person and their suffering. Never try to take charge of the situation. You should not let the person convince you that it is not serious, or that they can handle it on their own. However, if you think someone might be having suicidal thoughts, and you are unable to ask them, you should find someone who is able to talk to them.

You should try and have thorough information on the following:

- If the person has ever felt suicidal before, and if so, what happened then?
- If there is a planned method and specific way (e.g. quantity of poison) the person plans to use. This may indicate the seriousness of the suicidal intention.
- Whether the person knows anyone who has died by suicide and is, therefore, trying to solve their problems the same way.
- Are they suffering from a mental illness? This will give you the opportunity to reassure the person that you have no prejudice.
- Anything important in the person's life that may reduce the immediate risk of suicide (e.g. attachment to children).
- People they can turn to when they need help or support.
- Is there a change in a person's spiritual/religious beliefs (e.g. an increase or decrease in prayer, meditation or attending mosque, church, or temple) and, if this is the case, it would be useful to ask what is the impact of it on their wish/will to live?
- The person's cultural and religious beliefs regarding suicide, including, if the person believes in charms, evil spirits like jins, jadoo, taweez (amulets), or similar and, if so, if they believe those forces are affecting their current feeling.
- Who they can contact if they become suicidal again in future.

If the person is experiencing an episode of psychosis (a severe mental illness), you should avoid asking them if they have a 'mental illness' because this language may feel confrontational and may increase distress, instead ask if they are receiving help for any emotional or mental health problems, which may feel less stigmatizing.

If you think that the person does not want to talk to you or is uncomfortable interacting with you due to differences in age, gender, religion and/or cultural background, ethnicity, language or caste, ask the person if they would want to talk to a person more like themselves.

HOW SHOULDI TALK TO SOMEONE WHO IS SUICIDAL?

Letting them know you care

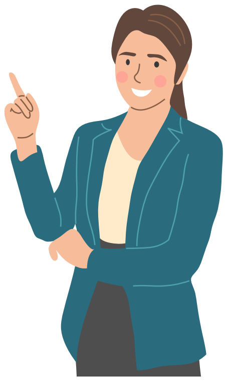

Tell the person that you care, want to help and do not want to lose them. It is important to reassure them that they are loved, worthy and would be missed. Give the person your full attention and reassure them that you want to hear whatever the person has to say and give them time to get to the topic about their suicidal thoughts (see the BOX 1 on Active listening tips). Ask the person open questions to understand what they are thinking and feeling. You would need to be patient and calm when the person is talking about their thoughts and feelings. Be supportive and understanding of the person and focus on the things that will keep the person safe for now, rather than the things that put the person at risk.

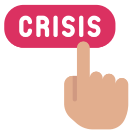

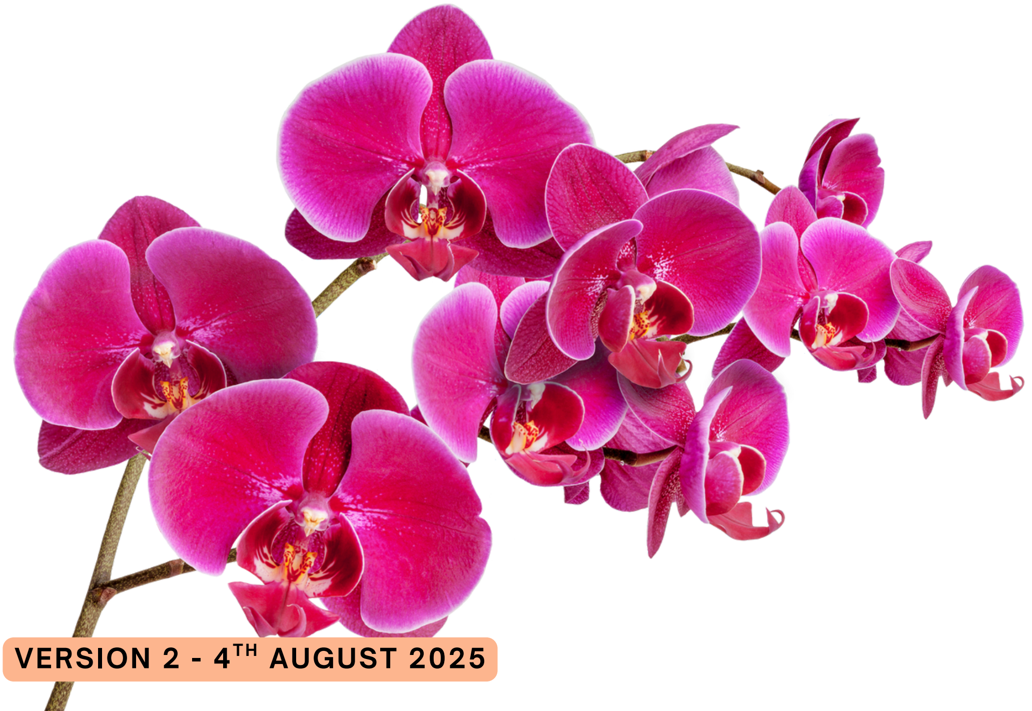

ORCHID - THE FLOWER OF WISDOM

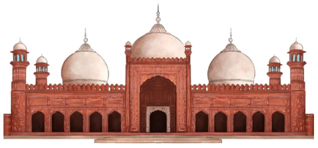

Box 1: Active Listening Tips

- Encourage the person to do most of the talking and listen to them without expressing judgment
- Let the person know that it is okay to talk about things that might be painful and express empathy for them (e.g. "I understand how you feel")
- Show them that you are interested and listening with full attention, e.g. by summarising what they are saying
- Clarify important points with the person to make sure you fully understand
- Ask simple questions, repeating these if necessary
- Avoid asking too many questions as it can provoke anxiety in the person
- You must also stay very conscious of your body language, making sure it is not communicating a lack of interest or negative attitude.

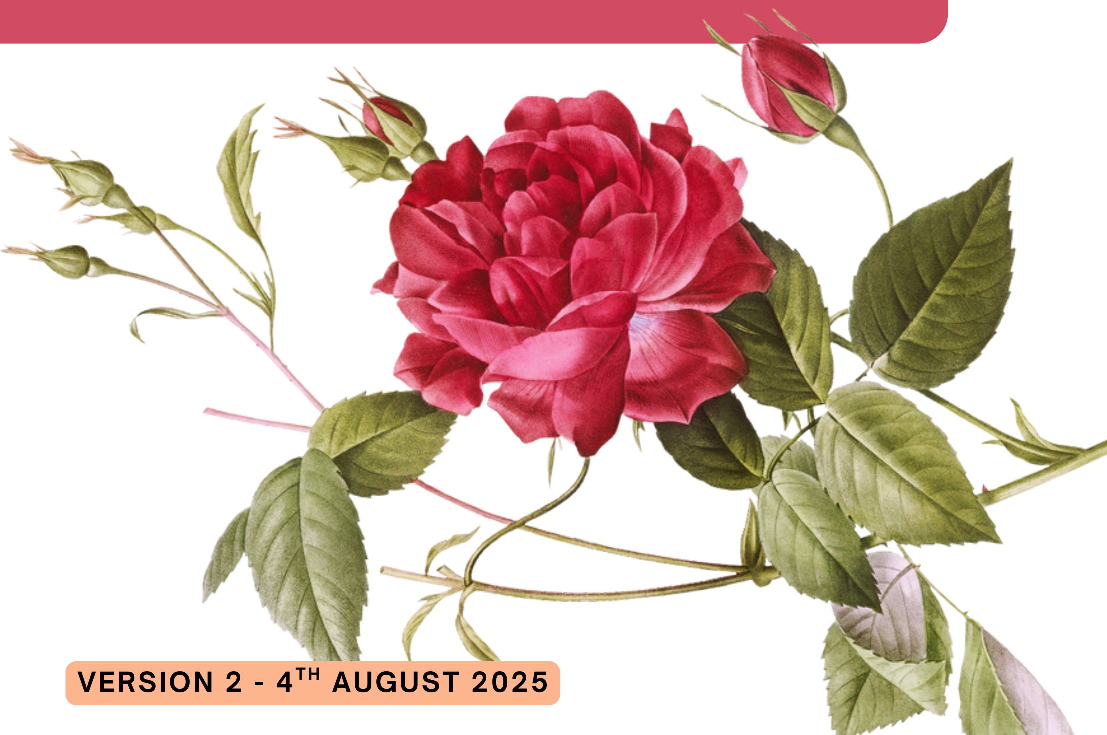

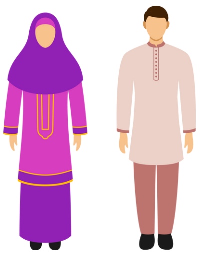

HOW CAN I OFFER REASSURANCE?

Tell the person that it is okay to feel the way they do and offer hope by reassuring that their feelings are temporary and highlight the fact that the person is still alive, and talking to you about their feelings means that they are not quite sure about suicide. Tell them that help is available and things will get better. Reassure them that the feelings they are experiencing may have been triggered by a mental health problem that can be treated. Try convincing them that you understand how badly they feel, and you are there for them and want to help.

Emphasise on their personal strengths, qualities, 'good and/or positive things' in their life, their dreams or goals and encourage them to stay alive so that they can make them come true. This will instil hope for the future and give them an opportunity to think about reasons to live.

It is important to normalise the situation by comforting them that we all go through tough times, and that it is not a crime, a sin or shame to feel suicidal. You may point out that such thoughts are common, many people have them at some stage in their lives and suicidal thoughts need not to be acted on. Tell them that it is possible to get help. Ask about what/who has supported them in the past and whether these supports are still available. Encourage the person that recognising the need for support and reaching out for help is the first step to feeling better, and acknowledge the courage required to share such feelings, and thank them for sharing their feelings with you.

HOW TO REACT WHEN A PERSON EXPRESSES SUICIDAL THOUGHTS?

Suicidal thoughts are often a desperate call for help and an attempt to escape from problems and distressing feelings. Instead of distracting the person (such as by saying "let's go out"), allow them to talk about difficult emotions and thoughts: they may feel relief at being able to do so and you should listen to what they say without agreeing or disagreeing with their behaviour or point of view. While doing so, you should be able to differentiate people with suicidal intentions that favour company in silence, and people with suicidal intentions that favour talking over silence. Be awarethat it is unhelpful to a person when their problems are being compared with others and to challenge them to realise their suicide plan.

Validate their thoughts and feelings and acknowledge that these may be hard to talk about. However, it is common to feel panic or shock when someone discloses thoughts of suicide. Avoid expressing negative reactions to suicidal thoughts such as judgement, shock and panic (BOX 2 - What to avoid when talking to the person). You ought to appear calm, confident, and empathic. This may have a soothing effect. If you have a close relationship with the person, you should show your care in a culturally appropriate manner by hugging them or holding their hand (with consent of the person) if both are of same gender as hugs can alleviate the person's psychological burden (see BOX 3). You should keep in mind gender sensitivity, as you do not want to further complicate matters by approaching in an inappropriate manner.

Allow the person to talk about their reasons for wanting to die. Also encourage them to discuss their reasons for living to make them realise that they are considering both options. Emphasise that living is always an option for them. It is also important to ask about issues that affect the immediate safety of the person who is suicidal and, if there is a crisis, you should say whatever you feel you need to in order to help them decide against suicide. If the person is at a point of despair, you should take control and be directive in ensuring their safety.

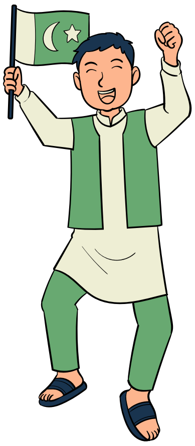

Box 2

Box 2:
What to avoid when talking to the person?

- Argue or debate with the person about their thoughts of suicide
- Discuss with the person whether suicide is right or wrong
- Tell the person to “Just be patient” as they might feel not understood
- Minimise the person's problems
- Give glib 'reassurance' such as 'don't worry', 'cheer up', ‘you have everything going for you' or 'everything will be alright'
- Interrupt with stories of your own
- Attempt to give the person a diagnosis of a mental health problem
- Discuss any mental health problems experienced by the person, focus instead on the reasons behind the suicide crisis
- Use guilt or threats to prevent suicide (e.g. do not tell the person that suicide is a sin and they will go to hell or ruin other people's lives if they die by suicide)
- Take any hurtful actions or words of the person personally
- Give advice as the person might not like to be advised
- Give judgmental spiritual advice (e.g. killing yourself is a sin, killing yourself means that you are becoming unthankful to Allah) and talk judgmentally about religion (e.g. to try to make them feel guilty), rather focus on other reasons to stay alive (e.g. parents and children)
- Touch (e.g. hug or hold hands) the person without their permission, unless you have a close personal relationship and/or are of the same gender.

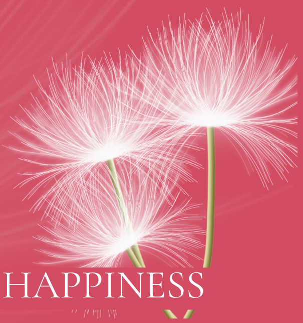

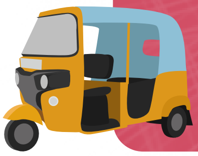

Box 3: Gender – specific considerations

- Be aware of different risk factors for a woman, such as domestic violence, postnatal depression, and interpersonal conflicts, and for a man, such as alcohol misuse and substance abuse, and financial difficulties
- Be aware that females are more likely to discuss physical complaints with no apparent physical source when in fact they are having suicidal thoughts
- Be aware that some males may be less likely to express their emotions and open about suicidal intentions
- Be aware that with females it is particularly important to discuss relationships issues, sexual interactions and related concerns in a sensitive manner
- Be aware that men may not openly disclose previous suicide attempts and may instead state, for example, that they had an "accidental overdose of medication or poison"
- Be aware that females from some cultural backgrounds may not be able to independently seek professional help and therefore family members must be involved
- Be aware that increased expression of emotions in males, such as crying or aggressive behaviours, could indicate suicidal risk

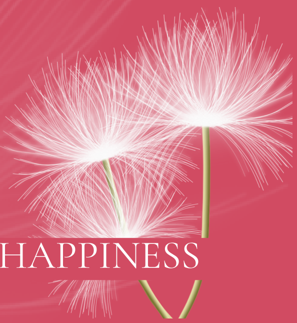

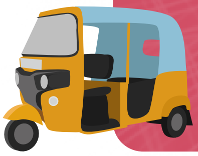

How can I assess the urgency of the situation?

It is important to take expressions of suicidal thoughts seriously and act on these, not dismissing them as 'attention seeking'. It is important for you to recognise the suicide warning signs (including number and nature of them), and major risk factors for suicide (e.g. recent stressful event, previous suicide attempt) to determine the urgency of taking actions. To explore this, ask the person how things are at home and at work/school, and if there have been changes in their employment, social life, or family.

Moreover, you should ask the person about their intentions to take their life as opposed to vague suicidal notions such as "what's the point?" or "I can't be bothered going on". Asking about a plan for suicide is also important. You should not let the person convince you that it is not serious or that they can handle it on their own. Rather, talk to them about current resources of support with which to help the person at risk, such as a mental health professional (e.g. psychiatrist, psychologist or school counsellor), another health professional, helpline (e.g. Umang), a friend, a family member, tribal/family elder, teacher, and/or a spiritual/religious leader. While assessing the urgency of taking action (e.g. emergency or mental health referral), you should try to get information about the person's mental health (e.g. presence of any disorder such as psychosis or bipolar).

ASKING ABOUT OTHER FACTORS THAT CONTRIBUTE TO THE RISK OF SUICIDE IN PEOPLE IN PAKISTAN

Be aware that there are certain groups of people who are more at risk for suicide such as:

- The elderly who are chronically ill and living alone
- Those with a diagnosis of severe mental illness (such as chronic depression)
- Users of alcohol or other hard drugs
- Those who have received mental health treatment in the past
- Any family history of mental health problems or suicide
- Those who have made a suicide plan in the past
- Those who made a suicide attempt in the past (be aware that there are many more suicide attempts than completed suicides). To get this information you should ask significant others e.g. family members, close friends or religious leader such as Imam-e-masjid
- More males die by suicide than females, but more females make non-fatal suicide attempts (however, the number of fatal attempts is increasing among females).

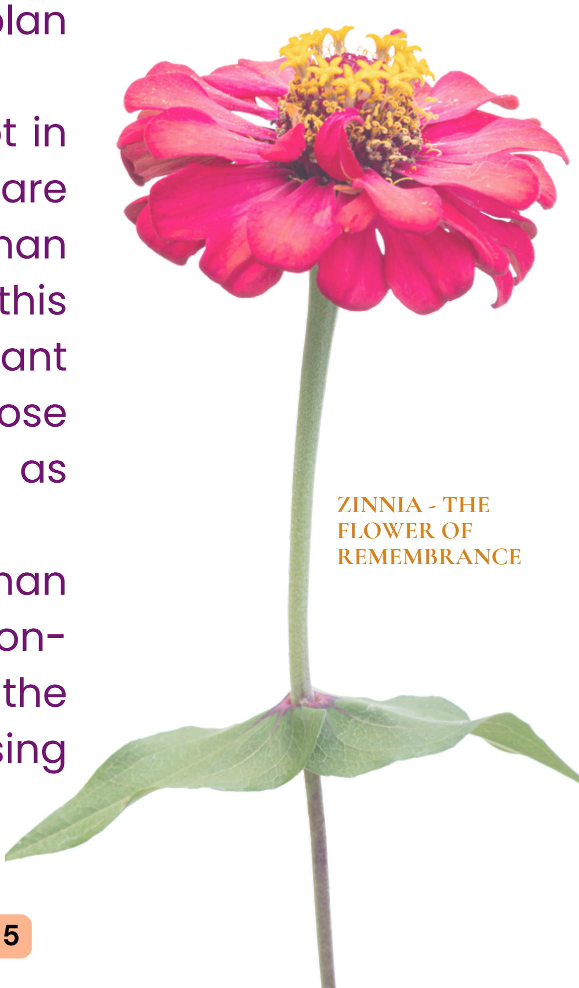

ZINNIA - THE FLOWER OF REMEMBRANCE

HOW TO PROVIDE INITIAL ASSISTANCE TO MAKE THE PERSON SAFE?

If you have established that the person is suicidal, you should work collaboratively with the person to ensure their safety, rather than acting alone. Make sure that you do not leave someone feeling suicidal on their own. This does not mean that you need to always be with them, rather check on them regularly. At the same time, it is also important that you should not put yourself in any danger while offering support to the person.

You must discuss with the person what actions they can take to get help and encourage them to seek appropriate professional support as soon as possible (i.e. see a mental health professional, a school counsellor or someone at a mental health service). You should know the phone numbers of suicide hotlines (mental health helpline, e.g. Umang, or the helpline of Aman Foundation or the helpline number by the Department of Health Pakistan; in case of adolescents, talk to a school counsellor, when available, emergency services, and mental health professionals).

Other than professional help, try to assist the person with the help of someone with whom they have a close relationship. Ask if they would like you to contact someone for them, such as a friend, family member, or trusted religious leader (such as a trusted Imam).

If you suspect any of the following situations, it is crucial to act quickly:

- An immediate risk of the person acting on suicidal thoughts
- The inability of the person to stay safe
- The person has a specific suicide plan
- The person is known to have a diagnosis of a mental illness (particularly of a psychotic disorder)
- The person has attempted suicide in the past.

HOW CAN I HELP THE PERSON WITH PROBLEM-SOLVING?

To reduce the risk of suicide, ask about the problems the person is facing and how you can help to solve their problems. By discussing specific problems, you should dispute the idea that suicide is the best or most viable solution and help the person work out ways of dealing with the difficulties that seem insurmountable. Assist the person in identifying available resources, suggesting solutions and a plan of action. This will indicate that there are other alternatives aside from suicide.

Rather than providing a solution to the person's problems and taking on their responsibilities, help the person put their problems into perspective by reminding them that other people may also face problems but still choose to live. As a problem-solving strategy, suggest things to distract the person from their suicidal thoughts, especially things which are relatively easy to do and will give them a sense of control and achievement. Help the person with positive and practical tasks. This can give the person a chance to spend some time dealing with their situation or give them a chance for some rest. Offer them practical support in making plans or setting goals for the future.

You should also educate the persons' family members, friends, their religious/spiritual leaders or significant other people about the suicide warning signs, risk and how they should assist the person.

HOW CAN I HELP A PERSON TO PASS TIME DURING A CRISIS?

Ask the person to postpone the decision to suicide and develop a list of pleasant activities they normally enjoy that they can do on their own or with you. You can suggest the following activities:

- Cooking a favourite meal
- Watching a movie or listening to music
- Relaxing activities, such as taking a hot bath, going for a long walk or reading something enjoyable
- Physical activity, such as going for a walk or a jog
- Spiritual and/or religious practices such as to pray, Wird/Zikr (recite supplication), Ijtema (religious gatherings), etc.)
- Involve trusted significant others in activities with the person to pass time
- Spending time with their significant others (e.g. family, friends, or religious leaders)
- Any other activity that has been found in the past to help them cope or that they enjoy.

If the person wants to be left alone, and can assure you of their safety, you should agree.

HOW CAN I HELP THE PERSON IN UNDERSTANDING THE CONSEQUENCES OF SUICIDE?

Encourage the person to consider the consequences of taking their own life including the effect it may have on the people they care about, for example, their family members. Discuss suicide as a possibility rather than an unthinkable act and remind them that suicide is a permanent solution to a temporary problem. Make the person aware about the possible consequences the suicide attempt could have on their health if they survived it, such as how overdosing can lead to messy, painful and long-drawn-out consequences (slow poisoning).

DEVELOPING A SAFETY PLAN WITH THE PERSON

In developing a safety plan for the person, fully engage them. Only make a safety plan with someone you know well. The plan should focus more on what the person should do rather than what they should not do. Make safety plans to ensure their safety for the next 24, 48 and 72 hours.

Such a plan should include:

- What needs to be done, who will be doing what, and when will it be carried out?
- An agreement that the person will not attempt suicide
- A list of safety contacts to be kept by the person, and an agreement/promise from them to call someone when they are feeling suicidal
- 24-hour safety contacts (such as the person's doctor or mental healthcare professional, a suicide helpline or crisis line, school counsellor in case of adolescents, as well as friends and family contact members) who will help in an emergency
- An agreement that the person will not use any alcohol or other drugs
- If the person is addicted to alcohol and drugs, in a safety plan do not remove what is their daily dose, meanwhile look for professional help for their alcohol or drug dependence
- Safety plan should not suggest any activity and location to be visited that could potentially trigger the person.

Make sure that the safety plan is kept somewhere accessible to the person. You should not assume that a safety plan is sufficient to keep the person safe, however, if the person refuses to make a safety plan, it would not be safe to leave them alone for any long period of time. Make sure someone stays close to the person(in the same room or in visual contact) and get outside help immediately (e.g. professional help).

WHEN SHOULD I NOT MAKE A SAFETY PLAN?

You should not use a safety plan with a person:

- Who has been diagnosed with severe mental illness (e.g. a psychotic disorder)
- who is using drugs or alcohol or
- who you do not know well.

IF THE PERSON IS RELUCTANT OR REFUSE TO GET PROFESSIONAL HELP

You must keep on convincing the person that they need help during the suicidal crisis and will not be judged or blamed for seeing a mental health professional. It is also important to identify their significant others (e.g. family or friends who are close to the person), let them know the situation and ask them to accompany the person to an emergency health service provider.

If the person is willing to seek professional help for their suicidal thoughts, you should help them plan what they will say and seek an agreement with them about the timeframe within which they will contact a specific person (who can help). If the person does not want to talk to someone face-to-face, you should call a mental health centre, or a crisis telephone helpline, and ask for advice, without letting the person know. Find out information on the resources and other services available for the person and encourage the person to contact a suicide prevention helpline. You may also have to call the police, but only when their intervention is necessary (e.g. the person is about to jump from a building) as suicide was an illegal act in Pakistan until recently and inform them that the person is suicidal to help them respond appropriately.

If despite all your efforts, the person refuses the professional help, you can do either of these two: contact the emergency service/helpline number without letting the person know or contact a health professional the person already knows and trusts, if there is one.

IF THE PERSON HAS THE MEANS TO CARRY OUT THEIR SUICIDE PLAN

If the person has potentially harmful items available, such as a weapon, gain the person's trust before removing the means of suicide and then remove access to these items, only if it is safe to do so. Dispose of them right away (i.e. flush pills and poison down the toilet, handgun to the police, throw away razors or knives).

You should pay attention to your own safety as well.

If the person does not agree to give you the weapon, you should try to take these things secretly. If this is not possible, you should remove yourself from the situation as your own safety is also hugely important. You should seek help from family members, neighbours or others to remove it; phone an emergency number without letting the person know and contact the police and inform them that the person is suicidal to help them respond appropriately. In case the discussion about suicide occurs over the phone, encourage the person to keep the weapon/dangerous objects out of reach and contact an emergency number so that the person is not alone.

In case of other means of suicide, such as prescribed medications, you should measure out prescription medication so that the person only has a certain amount available (e.g. couple of days' worth). Even after using all these strategies, you must keep in mind that you may not be successful in preventing suicide.

WHAT IF THE PERSON WANTS ME TO KEEP THEIR SUICIDAL INTENTION AS SECRET?

Never agree to keep the person's suicidal plans a secret and try to convince them that it is better to not keep their suicidal intentions a secret but involve someone else (e.g. a professional, a trustworthy friend or a family member, teacher, religious leader, a school counsellor, when available, in case of adolescents). You should involve them in making decisions about who else should know about their suicidal crisis. If the person does not want you to tell anyone about their suicidal thoughts, you should not agree and explain why, for instance, "I care about you too much to keep a secret like this" or "You need help, and I am here to help you get it".

To ensure confidentiality, treat everything the person says in complete confidence except their suicidal thoughts. However, if the person (including adolescents) asks you to promise to keep the discussion about suicide a secret, you should tell them that you may need to breach their confidentiality to ensure their safety and tell someone else anyway if needed. Ask for help from the person's relatives, friends or housemates to ensure the person does not have access to weapons, poisons, or other means for suicide. Call a mental health centre or crisis helpline and ask for advice on the situation or get the person to phone an emergency number (i.e. emergency services, a suicide prevention helpline/mental health helpline, emergency mental health services, school counsellor).

You should seek the permission of the person to contact their regular doctor/GP or mental health professional (e.g. psychiatrist) or other professional about their concerns and call them right away. In addition, if the person does not want you to contact anyone and you feel they might be at immediate suicide risk, you should personally take the person to the nearest safe place (e.g. hospital emergency department).

In certain situations, (e.g. if the person cannot commit to stay safe, or they have a specific plan), it is possible that you may have to contact emergency services without letting the person know. In these circumstances, it is possible that the person may express anger and feel betrayed by your attempt to prevent their suicide. However, you should stay calm, keep on listening the person non-judgmentally, and talk to them in a friendly tone. During crisis, help the person understand that they have control over their suicidal thoughts, and refer to their spiritual/religious beliefs (but not judgementally and with guilt) to prevent the person from taking their life.

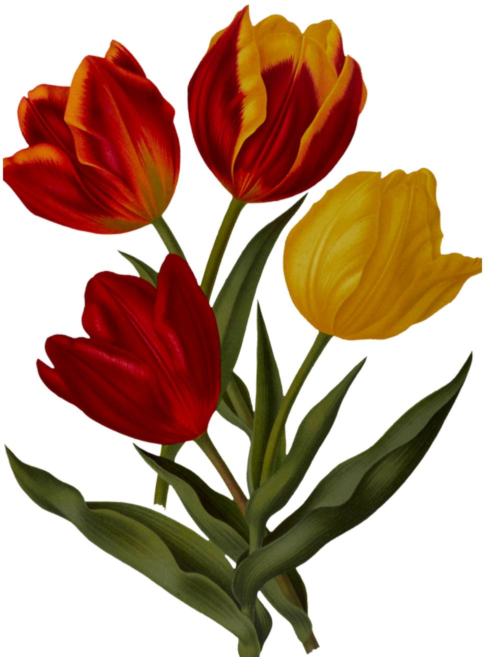

TAKE CARE OF YOURSELF

It is important to prioritise your own well-being when assisting someone who is feeling suicidal. Providing support in such situations can be emotionally draining. Ensure taking care of your own physical and emotional needs. If you are unable to fulfil this role, help the individual identify someone else who can assist them during the crisis.

Thank you for your help in saving lives and making this word a more compassionate place to live in.

AN IMPORTANT NOTE:

Purpose of These Best Practice Guidelines

These best practice guidelines are a general set of recommendations developed to help members of the public in offering initial assistance to individuals in Pakistan who may be at risk of suicide. The role of the first aider is to provide support to the person until they receive the appropriate professional help or until the crisis is resolved. Each individual is unique, and it is important to tailor your support to that person's needs. These recommendations, therefore, may not be appropriate for every person who may be at risk of suicide.

DEVELOPMENT OF THE BEST PRACTICE GUIDELINES

This best practice guidelines were developed through a detailed research process called Delphi method under the leadership of Prof Erminia Colucci, Department of Psychology, Middlesex University London, UK, Prof Nasim Chaudhry, Pakistan Institute of Living and Learning, Pakistan and Prof Nusrat Husain, The University of Manchester, UK, with assistance from Dr Tayyeba Kiran, Prof Imran B Chaudhry, Samia Shahid, Sehrish Tofique, Zaina, Tahira Khalid, Dr Sehrish Irshad, Ayesha Khaliq, Suleman Shakoor, and Atta-Ur-Rahman.

The Delphi method collected the expert opinions of a panel of experts with professional and lived experience with suicide and suicide prevention in Pakistan about how to help someone who may be at risk of suicide through questionnaires and focus groups. The questionnaire used at the initial phase of this Delphi study was based on the questionnaires used for the suicide prevention guidelines for Asian countries and for migrant and refugee populations led by Colucci, E. and available at https://doi.org/10.1080/18387357.2018.1469383. More details about the methodology can be found in the accompanying scientific article. Kiran T, Colucci E, Shahid S, Tofique S, Shakoor S, Imam Z, Husain N, Chaudhry N. Co-developing suicide prevention guidelines for Pakistan: a mixed-methods Delphi consensus study. BMC Public Health. 2025

Although these guidelines are copyrighted, they can be freely reproduced, made available online or electronically, for non-profit/commercial purposes provided the source is acknowledged.

ACKNOWLEDGEMENTS

We want to acknowledge all the experts including experts by lived experience for their participation in the Delphi process and the research team, in particular Dr Tayyeba Kiran and Prof Erminia Colucci.

We also want to acknowledge the funder, the Medical Research Council /DFID/NIHR programme (MR/R022461/1), and the Pakistan Institute of Living and Learning.

The funder has no role in developing the content of this best practice guidelines, designing and publishing.

If you are interested to know more about the Pakistani guidelines or to receive the scientific article, please contact:

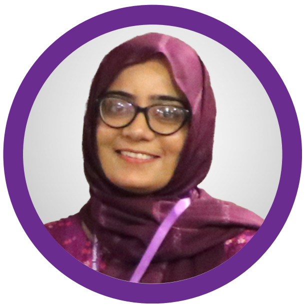

Email: tayyaba.kiran@pill.org.pk

DR. TAYYEBA KIRAN

Assistant Director Research & Development
Pakistan Institute of Living and Learning

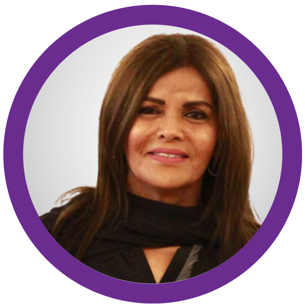

Email: nasim.chaudhry@pill.org.pk

PROF. NASIM CHAUDHRY

Chief Executive Officer
Pakistan Institute of Living and Learning,
Associate Director
Global Mental Health and Cultural Psychiatry,
Professor of Psychiatry
Dow University of Health Sciences,

If you want to know more about the suicide prevention guidelines approach you can find a chapter in Colucci, E. & Lester, D. (2025) Suicide and Culture 2.0: Understanding the context (Hogrefe) and/or contact Prof Erminia Colucci

Email: erminia.colucci@mdx.ac.uk
