# Supplementary material for: Co-developing suicide prevention guidelines for pakistan: a mixed-methods Delphi consensus study
Source: BMC Public Health. 2025 Oct 21;25:3536. doi: 10.1186/s12889-025-23942-3 (PMC12538931; doi:10.1186/s12889-025-23942-3)
Supplement: Supplementary file 3 — Supplementary Material 3 [file 12889_2025_23942_MOESM3_ESM.pdf]

	SUICIDE FIRST AID GUIDELINES FOR PAKISTAN- INTRODUCTION
	**Purpose of the research**<br>The aim of this project is to develop guidelines for members of the public to provide first-aid to a person in Pakistan who is having suicidal thoughts or displaying suicidal behavior.<br>As a first step, we are seeking to find consensus on the key skills and knowledge that a person needs in order to help someone in Pakistan who is having suicidal thoughts or displaying suicidal behavior. The statements from this questionnaire that receive a high level of consensus will be included in the Suicide First Aid Guidelines for Pakistan. The guidelines may be used as the basis for suicide prevention training programs.
	**Your role**<br>You have been selected as a panel member for this study because you have (personal and/or professional) expertise in the area of suicide prevention for people in Pakistan. Your task is to rate the statements presented in this questionnaire according to how important you believe they are to the aims of suicide first aid and the role of the first aider.<br>The role of a Mental Health First Aider for suicide is to recognize and help a person who is in a suicidal crisis, for example, is having suicidal thoughts or displaying suicidal behavior. The first aid is given until appropriate professional treatment is received, or the crisis resolves and the person decides against suicide. It does not include counselling or therapy.
	**How was this questionnaire developed?**<br>The statements in the following questionnaire were derived from suicide prevention materials, published help books, websites, online fact sheets and scientific journal articles and previous Suicide First Aid Guidelines research (see https://movie-ment.org/suicide-first-aid-guidelines/).<br>There will be warning signs and actions that would be appropriate for members of the public in Pakistan, which are not included in this questionnaire, and there will be warning signs and actions that may be appropriate in some countries but not in your context. At the bottom of each page, there is space for you to add suggestions. Please consider the cultural, social and religious/spiritual context in Pakistan, and add some relevant suggestions on each page.<br>The more you add to this questionnaire, the more relevant and useful the guidelines will be for Pakistan. Some of the statements may seem contradictory or controversial; however, we included them because they reflect the wide range of people’s beliefs about intervention and care. It is important to note that we do not necessarily agree with these statements; however, we included them because we do not believe that we should decide what the best practice is for suicide first aid. Rather, we have invited you to be a member of the expert panel to help select the statements to develop a set of guidelines that reflect experts' experience across the field of suicide prevention.

	**Other instructions to note**<br>This questionnaire should take approximately 60 minutes to complete. When completing this questionnaire, you will read statements describing possible warning signs and actions that the first aider can take to assist a suicidal individual. You will be asked to rate how important each item is as a guidance for a first aider. Please rate as "essential" or "important" those items that you feel should guide most people, most of the time, when assisting a suicidal person.<br>Please be aware that once you have logged on and started responding, you may save your answers at any time by completing a page and clicking "Next" at the bottom. This marks your page as complete and you may continue at another time from the next page. Please make sure that you always log back in using the same email link, otherwise the software will fail to recognize your code and access previously saved responses.<br>Remember that this survey is linked to your own email so if you would like to help us in recruiting other experts (professionals and suicide survivors/people with lived experience), please contact the research fellow on the project, Dr Tayyaba Kiran (tayyaba.kiran@pill.org.pk). Your help in reaching other relevant participants (i.e. experts by lived experience and/or profession) for this study is much appreciated!<br>We would like to thank you for your time and effort and encourage you to provide us with suggestions regarding the development of the guidelines and future suicide prevention programs in Pakistan.<br>**Best wishes**<br>**Prof. Nasim Chaudhry, Pakistan Institute of Living and Learning & Prof. Erminia Colucci, Middlesex University London (Project Co-Leads)**

DEFINITION OF THE TERMS USED IN THE QUESTIONNAIRE

Throughout the questionnaire, we use the terms ‘the suicidal person’ or ‘the person’ to refer to the person experiencing suicidal thoughts or displaying suicidal behavior, and ‘the first aider’ to refer to the person providing assistance.

Mental health problem is a broad term that includes developing mental illness, symptoms of a diagnosable illness, substance use, and adverse life events which are having an impact on functioning. A mental illness is a mental health problem that has been diagnosed by a mental health professional, that affects a person’s thinking, emotional state and behavior, and disrupts a person’s ability to work or carry out other daily activities and engage in satisfying personal relationships. ***Psychosis*** refers to symptoms in which there is misinterpretation and misapprehension of the nature of reality, such as hallucinations, delusions, or thought disorders.

Emergency services refers to the best services available at short notice for the situation in the area. ***Emergency mental health services*** refers to mental health services that are available at short notice in the area, and could include a crisis team or a suicide helpline.
A ***Safety plan*** is an agreement between the suicidal person and the first aider that involves actions to keep the suicidal person safe.

A **Suicide lived experience advocate** is someone supporting suicide prevention who has a previous personal experience of suicidal ideation or attempts, or loss of someone close to them through suicide, or has assisted someone at suicide risk.

Please be aware that the statements in this survey only apply to first aid given by adults. However, there are some statements specifically about the support that an adult could provide to an adolescent. These items are included in cases where the support provided to an adolescent might be quite different to the support provided to an adult.

The term ***adolescent*** refers to a young person aged between 12 and 18 years (nominally the high school years).

DEMOGRAPHIC AND SUICIDE PREVENTION ROLE INFORMATION

Please provide your details below to help us with describing the overall research demographics

Sex	Male	Female	
Your age (in years)			

	Which best describes your main area of expertise?<br>Please note: Only people who have professional (as clinicians or researchers) and/or personal (either themselves or someone close to them) experience of suicide for people in Pakistan are eligible for this study. [you may tick more than one answer]					
	a. Suicide Prevention Professional (Clinician, for example psychiatrist, psychologist, GP, nurses)					
	b. Community Leader (police, religious leader, community leader, other stakeholders)					
	c. Suicide survivor (i.e. person who has tried to kill themselves and/or significant others of someone who tried to kill themselves or died by suicide).					
	**Please name the mental health and/or suicide prevention organizations you are affiliated with and your role within each organization. If none, please write N/A.**					
	**Please provide details of your primary place of work**					
	**City/Town**					
	**Province**					
	**Which of the following best describes your role?**	**Psychologist**	**Psychiatist**	**Social worker**	**GP**	**Nurse**
		**Occupational therapist**	**member from non-profit organization**	**Community health worker**	**Religious scholar**	**Traditional healer**

		**service user**	**carer**	**Other. Please specifi**		
	**SECTION 1. IDENTIFICATION OF SUICIDE RISK**					
	This section contains statements about identifying the severity of the risk of suicide in a person in Pakistan.<br>NOTE: If the person has already acted on their thoughts of suicide, this should be treated as a medical emergency.<br>Suicide warning signs<br>Warning signs for suicide are defined as the earliest detectable indication of increased risk for suicide in the near future (i.e. within minutes, hours or days).					
	**Would you consider the following to be warning signs of suicide in Pakistan?**	**Definitely yes**	**Probably yes**	**Not sure**	**Definitely no**	**Probably no**
1	Threatening to hurt or kill themselves.					
2	Looking for a way to kill themselves (e.g. seeking access to pills or poisons, weapons or other means), including asking information about possible suicide methods (e.g. "would 100 mg of this kill me?" or “would I die if I jumped from that building?" or “would rat pills or wheat pills kill me?" or "would this much bleach poison me?”).					
3	Talking or writing about death, dying or suicide (including making unexpected jokes about these topics, or leaving a suicidal note such as on social media (whatsapp, instagram etc), or a poem or letter).					
4	No longer talking or writing about death, dying or suicide (including no longer making jokes about these topics).					

5	Unusual engagement with suicide related material such as movies, games, news, etc.					
6	Expressions of hopelessness.					
7	Expressions of rage, anger, aggressiveness and/or seeking revenge.					
8	Describing themselves as a burden to others or expressing feelings of guilt or shame (e.g. stating that others will be better off without them).					
9	Writing stories that have the protagonist (hero, leading character) die in them.					
10	Feeling too lazy to do anything.					
11	Lying down all the time.					
12	Keep on looking for dark places.					
13	Becoming more sensitive, easy to be angered.					
14	Not trusting anyone, including thinking that everyone is insincere or doubting everyone's sincerity.					
15	Dramatic change of habits.					
16	Acting recklessly or engaging in risky activities, seemingly without thinking.					
17	Expression of feeling trapped, like there is no way out.					
18	Starting or increasing cigarette, alcohol or drug use.					
19	Withdrawing from friends, family or society, including locking oneself in the house or in a particular room.					
20	Telling significant others that they want to end their life.					

21	Isolating themselves from interacting with others.					
22	Unusually high levels of anxiety or agitation.					
23	Sleep disturbance – inability to sleep, or need to sleep all the time.					
24	Dramatic change in behavior, mood, appearance.					
25	Sudden increase of restlessness.					
26	Sudden recovery from depressed/sad mood.					
27	Sudden or dramatic increase in depressed/sad mood (including crying more than usual or lack of smiling).					
28	Engaging in self -injurious behavior such as cutting, burning themselves, poisoning (e.g. drinking cleaning agent (such as bleach, phenyl) or mosquito repellent (such as mospel), or insecticide (such as wheat or rat pill etc.) or hitting their head against the wall.					
29	Rejecting or stopping life-saving medical treatments/medications.					
30	Giving away valued possessions and getting affairs in order, including asking others to take on responsibility for the care of people or pets.					
31	Expressing a lack of reasons for living, or having no purpose in life.					
32	A lack of interest in or plans for the future.					

33	Significant change (increase or decrease) in the level of religious interest or involvement in religious activities (e.g. praying, reading religious books, spending more time in mazar).					
34	A sudden change of their religion, faith or belief (e.g. from Islam to another religion) or a change in sect (i.e. from Ehl e Sunnah to another sect).					
35	Increased preoccupation with death, dying and/or the after life.					
36	A lack of response (e.g. 'freeze reaction') in a situation of crisis.					
37	Decrease in attention or concentration.					
38	Decrease in their job outcomes or school performance (e.g. receiving lower marks).					
39	Sudden change in behavior or mood with calm and contended thoughts and actions after a period of anxious or low mood.					
40	Increased expression of affection or anger shown towards children.					
41	Expressing interest in renouncing lay life and joining a religious institution (e.g. joining a Tableeghi jamat or Ijtamah).					
42	Refraining in seeking medical assistance for physical complaints.					
43	Expressing recurrent physical complaints with no clear physical source (e.g. burning sensation, body aches, stomach related issues, heaviness, fatigue).					

44	Suddenly developing a strong fear about something.					
45	Decrease of appetite and weight loss not explained by other factors (e.g. diet or sickness).					
46	Refusing to eat for days.					
47	Daring someone to kill them (e.g. "just shoot me").					
48	Putting oneself in a situation with high risk of being killed (e.g. confronting armed officers, like the police).					
49	Longing for deceased loved ones, including being eager to see someone who is no longer alive or increasing visits to their burial site (grave yard "Qabristaan").					
50	Staying still and staring (e.g. to people walking by).					
51	Showing signs of lack of energy or enthusiasm.					
52	Feeling rejected by others.					
53	Feeling worthless or that their life is worthless.					
54	Having the desire or hope that they will die (including praying that God may take their life).					
55	Person expresses in words or action that suicide is the only solution to their problems.					
56	Person expresses in words or action the loss of interest of the things they used to be interested in.					
57	Having feelings of extreme dislike or hatred of oneself.					

58	Having strong sense of feeling alone and/or cut off (feeling lonely or isolated).					
59	Making arrangements for one’s own funeral or buying items generally used for funeral (e.g. Islamic caffeine or fix/buy a place in graveyard).					
60	An important warning sign is contacting people (e.g. family members and/or people they have not spoken to in a long time) to say goodbye, making amends or asking for forgiveness.					
61	An important warning sign is stating that they want to disappear or disappearing.					
62	An important warning sign for suicide is a person who states that their 'time has come' and/or that it is time to rest.					
63	An important warning sign for suicide is a person starts giving advices to others (e.g. about life) in a way that is atypical of them.					
64	An important warning sign of suicide is if a person is engaging in self-injurious behaviours such as cutting or burning themselves.					
65	An important warning sign for suicide is if a person is expressing in words or actions that they are unable to find an alternative solution to a problem.					
66	An important warning sign for suicide is if a person expresses in words or actions a sense of guilt or self-blame for something that has happened.					

67	An important warning sign for suicide is if the person stops expressing themselves.					
68	Please use the space below to suggest at least three warning signs that you feel are important for the identification of suicide risk for people in Pakistan.					
68.1						
68.2						
68.3						
69	Do you have any comments on these statements? Is there anything you would like to add to this section? Please write your ideas in the box provided					
	**Things a First Aider has to consider before approaching the suicidal person (please note that for all following sections only items rated as "essential" or "important" will be included in the suicide first aid guidelines)**	**Essential**	**Important**	**Don't know**	**Unimportant**	**Should not be included**
70	During crisis, the person doesn't like to be advised.					
71	The first aider should be able to differentiate people with suicidal intentions that favor company in silence, and people with suicidal intentions that favor talking over silence.					
72	The first aider should not assume that the person will get better without help.					

73	The first aider should know that hugs can alleviate the person's psychological burden.					
74	The first aider should not give judgmental spiritual advice (e.g. killing yourself is a sin, killing yourself means that you are becoming unthankful to Allah).					
75	First aider does not always have to come in the form of physical presence, but can also help through the phone.					
76	The person does not like it when their problems are being compared with others'.					
77	The person does not like to be challenged to realize their suicide plan.					
78	The first aider should not assume that the person will seek help on their own.					
79	The first aider should be able to recognize the warning signs of suicide.					
80	If the suicidal person has already harmed themselves, the first aider should administer first aid and call emergency services, asking for an ambulance.					
81	The first aider should not assume that a person who self-harms is suicidal. If the first aider is unsure whether noticed wounds are from a self-injury or from a suicide attempt, the first aider should ask the person directly.					
82	The first aider should talk to other people who know the person to see if they also have concerns.					

83	The first aider should have suicide crisis resources on hand before starting a conversation with someone who might be having suicidal thoughts.					
84	The first aider should act promptly if they think someone is considering suicide.					
85	The first aider should choose a private place to talk to the person about their concerns.					
86	The first aider should choose a time to talk to the person when there is sufficient time to discuss their concerns.					
87	In case of immediate risk of suicide, the first aider should not wait to find sufficient time to talk to the person. This may cause delay or keep that person at risk without intervening.					
88	The first aider should understand that sometimes people consider suicide to be a better choice than being a burden to others.					
	**How to make the approach - The first aider should:**	**Essential**	**Important**	**Don't know**	**Unimportant**	**Should not be included**
89	Tell the person their concerns about them, describing behaviors that have caused them to be concerned about suicide.					
90	Avoid raising the topic of suicide with the person during an argument or if they are really upset because this may end up getting a bad reaction and distancing them.					
91	Be aware of their attitudes about suicide and the impact of these upon their ability to provide assistance, e.g. beliefs that suicide is wrong, forbidden in Islam or that it is a rational option.					

92	Demonstrate appropriate language when referring to suicide by using the terms 'suicide' or 'died by suicide'.					
93	Avoid using terms to describe suicide that promote stigmatizing attitudes, e.g. 'commit suicide' or refer to a suicide attempt as having 'failed' or been 'unsuccessful' or using judgmental words like ‘crazy’.					
94	Be aware that different cultures have different beliefs and attitudes about suicide (such as in our context both religious and legal aspects. Suicide is forbidden in Islam. It is still a medicolegal case in Pakistan).					
95	If the first aider is concerned about someone who is from a different religion and/or cultural background to their own, they should learn about their religious/cultural beliefs and attitudes towards suicide before approaching the person.					
	**Asking about suicidal thoughts**	**Essential**	**Important**	**Don't know**	**Unimportant**	**Should not be**
96	If the first aider thinks someone might be having suicidal thoughts, they should ask that person directly.					
97	The first aider should ask the person about suicidal thoughts, even if the first aider feels uncomfortable doing so.					
98	If the first aider has even a mild suspicion that the person is having suicidal thoughts, they should ask.					

99	If the first aider thinks someone might be having suicidal thoughts and feels unable to ask them, the first aider should find someone who is able to ask.					
	**The first aider should:**	**Essential**	**Important**	**Don't know**	**Unimportant**	**Should not be included**
100	Not avoid using the word 'suicide'. It is important to discuss the issue directly, without dread or expressing negative judgement.					
101	The first aider should know that it is more important to ask about suicidal thoughts than to be concerned about the exact wording.					
	**The first aider should be aware that:**	**Essential**	**Important**	**Don't know**	**Unimportant**	**Should not be included**
102	If a person is not suicidal, asking them cannot put the idea of suicide in their head.					
103	If a person is suicidal, asking them about suicidal thoughts will not increase the risk that they will act on these.					
104	If a person is suicidal, asking them about suicidal thoughts will allow them the chance to talk about their problems and show them that somebody cares.					
105	The stigma associated with suicide might refrain the suicidal person from disclosing suicidal thoughts and seeking help.					
106	The existence of method and specific modality (e.g. quantity of poison) the suicidal person plans to use could indicate the seriousness of the suicidal intention.					

107	If the first aider thinks someone might be having suicidal thoughts, they should ask that person indirectly at first (i.e. "Do you ever wish you did not wake up in the morning?") and then only ask directly if the person says yes.					
108	The first aider should ask the suicidal person if they know anyone who has suicided and if they are trying to solve their problems like they did.					
109	If the first aider thinks someone might be having suicidal thoughts, they should begin the conversation by asking the person about how they are feeling.					
110	The first aider should ask about and allow the person time to discuss their negative feelings before asking about suicidal thoughts.					
111	When the first aider asks the suicidal person if they are suffering from a mental illness, they should reassure the person that they have no prejudice against people with mental illness.					
112	The first aider should try to determine whether there is anything important in the person's life that may reduce the immediate risk of suicide (e.g. attachment to children).					
113	The first aider should ask the suicidal person if there are people they can turn to when they need help or support.					
114	The first aider should respect the suicidal person and not try to take charge of the situation.					

115	The first aider should not let the suicidal person convince them that it is not serious or that they can handle it on their own.					
116	The first aider should ask the suicidal person if they have experienced a change in their spiritual/religious beliefs (e.g. an increase or decrease in prayer, meditation or attending mosque, church, or temple) and if this is the case, it would be useful to ask what is the impact, such as on their wish/will to live.					
117	The first aider should ask the suicidal person about their cultural and religious beliefs regarding suicide					
118	The first aider should explore the person's attitude towards suicide (e.g. "suicide is a heroic thing to do").					
119	The first aider should ask if the person thinks they are being punished by God (Allah) or a spiritual force for their wrong doings and therefore feel obliged to end their life.					
120	The first aider should ask if the suicidal person believes in charms, evil spirits like *jins, jadoo, taweez or similar* and, if so, if they believe those forces are affecting their current feelings.					
121	The first aider should ask if the person has felt suicidal before and if so, what happened in that occasion.					
	**If the person doesn't want to talk**	**Essential**	**Important**	**Don't know**	**Unimportant**	**Should not be included**

122	The first aider should understand that the person may not want to talk with them, and should offer to help them find someone else to talk to.					
123	If the first aider thinks the person is uncomfortable interacting with them due to differences in age group or gender, they should ask the person if they would prefer to talk to someone of the same age group or gender.					
124	If the first aider thinks the person is uncomfortable interacting with them due to differences in religion and/or cultural background, they should ask the person if they would prefer to talk to someone of the same religion and/or cultural background.					
125	If the first aider thinks the person is uncomfortable interacting with them due to differences in ethnicity, language or caste, they should ask the person if they would prefer to talk to someone of the same background.					
	**If the first aider feels unsuccessful in their approach**	**Essential**	**Important**	**Don't know**	**Unimportant**	**Should not be included**
126	If the first aider is unable to make a connection with the person, they should offer to help them find someone else to talk to.					

127	If the first aider feels uncomfortable interacting with the person due to differences in age group or gender, they should seek assistance from someone of the same age group or gender as the person.					
128	If the first aider feels uncomfortable interacting with the person due to differences in religion and/or cultural, they should seek the assistance of someone of the same religion and/or cultural background as the person.					
	**Reacting to expressions of suicidal thoughts**	**Essential**	**Important**	**Don't know**	**Unimportant**	**Should not be included**
	**The first aider should:**					
129	Know that it is common to feel panic or shock when someone discloses thoughts of suicide.					
130	Appear calm and confident in the face of the suicide crisis, as this may have a re-assuring effect for the suicidal person.					
131	Recognize and be respectful of the suffering of the suicidal person.					
132	React to expressions of suicidal thoughts with calmness and empathy.					
133	Avoid expressing negative reactions to suicidal thoughts, e.g. judgement, shock, panic, anger.					
134	Allow the suicidal person to discuss their feelings. A suicidal person may feel relief at being able to do so.					
135	Know that if the distressed person says they are not suicidal, they probably are not.					

136	If the person is at a point of despair, the first aider needs to take control and be directive in ensuring their safety.					
137	If the first aider clearly states that thoughts of suicide may be associated with a treatable disorder, this may instill a sense of hope for the suicidal person.					
	**If the person is experiencing an episode of psychosis (a severe mental illness)**	**Essential**	**Important**	**Don't know**	**Unimportant**	**Should not be included**
138	The first aider should avoid asking the suicidal person if they have a 'mental illness', instead asking if they are receiving help for any emotional or mental health problems.					
139	If the person is psychotic, the first aider may not be able to believe them if they say they are not suicidal.					
140	If the suicidal person says they are hearing voices, the first aider should ask if the voices are telling them to kill themselves.					
	**If the person is under the influence of drugs & alcohol**	**Essential**	**Important**	**Don't know**	**Unimportant**	**Should not be included**
141	If the person is using drugs or alcohol, the first aider may not be able to believe them if they say they are not suicidal.					
142	Please use the space below to suggest at least three first aid actions that you feel are important for the identification of suicide risk for people in Pakistan.					
142.1						

142.2						
142.3						
143	Do you have any comments on these statements? Is there anything you would like to add to this section? Please write your ideas in space below.					
	**END OF SECTION 1**					
	**SECTION 2. ASSESSING SERIOUSNESS OF THE SUICIDE RISK**					
	This section contains statements about assessing the seriousness of the person's risk of suicide in people in Pakistan. Please rate how important (from essential to should not be included) you think it is that each statement be included in the final guidelines.					
	**Assessing the urgency of suicide risk**	**Essential**	**Important**	**Don't know**	**Unimportant**	**Should not be included**
144	The first aider should take all thoughts of suicide seriously. The lack of a plan for suicide is not sufficient to ensure safety.					
145	The first aider should determine the urgency of taking action based on recognition of suicide warning signs.					
146	The first aider should know which are the major risk factors for suicide (e.g. recent stressful event, previous suicide attempt).					

147	The first aider should take expressions of suicidal thoughts seriously and act on these, not dismissing them as 'attention seeking' or a 'cry for help'.					
148	The first aider should ask the suicidal person if they are really serious or just looking for attention.					
149	The first aider should establish whether the person has definite plans and intentions to take their life as opposed to vague suicidal notions such as "what's the point?" or "I can't be bothered going on".					
150	If the suicidal person says that the situation is not serious or that they can handle it on their own, the first aider should respect this.					
151	The first aider should not let the suicidal person convince them that it is not serious or that they can handle it on their own.					
152	The first aider should ask significant others (e.g. family members, close friends or religious leader such as Imam-e-masjid) whether the person has made a previous suicidal attempt.					
	**Finding out about a suicide plan - The first aider should:**	**Essential**	**Important**	**Don't know**	**Unimportant**	**Should not be included**
153	Ask the suicidal person if they have a plan for suicide.					
154	Ask the suicidal person how they intend to suicide i.e. ask them direct questions about how, when and where they intend to suicide.					

155	Ask the suicidal person if they have decided when they will carry out their plan.					
156	Find out if the suicidal person has already taken steps to secure the means to end their life.					
157	Be aware that those at the highest risk for acting on thoughts of suicide in the near future have a specific suicide plan, the means to carry out the plan, a time set for doing it, and an intention to do it.					
	**Asking about other factors that contribute to risk of suicide in people in Pakistan**	**Essential**	**Important**	**Don't know**	**Unimportant**	**Should not be included**
158	The first aider should be able to recognize the person's level of suicide risk by the number and nature of warning signs.					
159	The first aider should be aware that there are certain groups of people who are more at risk for suicide such as elderly people who are chronically ill and living alone.					
	**The first aider should ask the suicidal person:**	**Essential**	**Important**	**Don't know**	**Unimportant**	**Should not be included**
160	Any visible (blindness, physically handicapped) or invisible disability (intellectual disability/mental retardation)					
161	If they have been using drugs or alcohol.					
162	If they have received treatment for mental health problems or is taking any medication.					
163	If they have received mental health treatment in the past.					

164	If they have ever known anyone who has died by suicide.					
165	About any family history of mental health problems or suicide.					
166	If they have ever made a suicide plan in the past.					
167	If they have ever made a suicide attempt in the past.					
168	How they are feeling right now.					
169	If they have told anyone about how they are feeling.					
170	How things are at home and work/school.					
171	If there have been changes in their employment, social life, or family.					
172	About current sources of support to help the person at risk such as mental health professional (psychiatrist, psychologist), another health professional, helpline (Umang), a friend, a family member, teachers, a spirtual leader/religious cholat etc. If there are people they can turn to when they need help or support .					
173	Please use the space below to suggest at least three first aid actions that you feel are important for assessing the seriousness of suicide risk among people in *Pakistan* .					
173.1						
173.2						

173.3						
174	Do you have any comments on these statements? Is there anything you would like to add to this section? Please write your ideas in the box provided.					
	**END OF SECTION 2**					
	**SECTION 3. INITIAL ASSISTANCE TO A SUICIDAL PERSON**					
	This section contains statements about the assistance to be first provided to the suicidal person in Pakistan. Please rate how important (from essential to should not be included) you think it is that each statement be included in the final guidelines.					
		**Essential**	**Important**	**Don't know**	**Unimportant**	**Should not be included**
175	The first aider should know the phone numbers of suicide hotlines (mental health helpline e.g. Umang or helpline by Aman Foundation, helpline number by Department of Health Pakistan), emergency services, and mental health professionals.					
176	The first aider should try to assist a suicidal person with whom they have a close relationship.					
177	The first aider should not put themselves in any danger while offering support to the suicidal person.					
178	The first aider should not leave someone who is feeling suicidal on their own.					

179	The first aider does not need to be with the suicidal person all the time, but should check on them regularly.					
180	The first aider should work collaboratively with the suicidal person to ensure their safety, rather than acting alone to prevent suicide.					
181	If the first aider suspects there is an immediate risk of the person acting on suicidal thoughts, they should act quickly even if they are unsure.					
182	The first aider should be prepared for the suicidal person to possibly express anger and feel betrayed by their attempt to prevent their suicide or help them get professional help.					
183	When talking to the suicidal person, the first aider should use the person's belief systems and values to encourage them to change their mind about suicide.					
	**If the person is suicidal - The first aider should:**	**Essential**	**Important**	**Don't know**	**Unimportant**	**Should not be included**
184	Discuss with the suicidal person what actions they should take to get help.					
185	Encourage the suicidal person to get appropriate professional help as soon as possible (i.e. see a mental health professional or someone at a mental health service).					
186	Seek the permission of the suicidal person to contact their regular doctor or mental health professional about their concerns.					
187	Take the suicidal person to the nearest safe place (e.g. hospital).					

188	Call a mental health centre or crisis telephone line and ask for advice on the situation.					
189	Call their doctor/GP, psychiatrist or other professional right away for the suicidal person.					
190	Take the person to a doctor/GP, psychiatrist or other mental health professional as soon as possible.					
191	Get the suicidal person to phone an emergency number (i.e. Emergency services, a suicide helpline/mental health helpline, emergency mental health services).					
192	Ask the suicidal person if they would like the first aider to contact someone for them, such as a friend, family member, or trusted religious leader (such as a trusted Imam).					
193	Phone an emergency number without letting the suicidal person know.					
194	Take the suicidal person to a hospital emergency department.					
195	Help the suicidal person understand that they have control over their suicidal thoughts.					
196	Remain calm and in control when communicating with a suicidal person.					
197	Consider the suicidal person's spiritual/religious beliefs and refer to these to try to prevent the person from taking their life.					
198	Listen non-judgmentally to the suicidal person.					

199	Be aware that suicidal people differ in their chosen suicide methods so they should pay attention to the presence of any sort of potential suicidal means (not just guns, rope, pills but also knives, any kind of poison, kerosene, bleaching agents, phenyl, rat pills and so on).					
200	Get rid of any sort of potential suicidal means (not just guns, rope, pills but also knives, any kind of poison, kerosene, bleaching agents, phenyl, rat pill and so on).					
	**If the person is suicidal - The first aider should not:**					
201	Offer false hope, or make unrealistic promises.					
202	Dismiss the person's feelings or compare their problems to the problems of others.					
203	Make the person feel guilty about wanting to die (e.g. by saying "aren't you ashamed to run away from life?").					
	**If the suicidal person can't commit to stay safe, the first aider should:**	**Essential**	**Important**	**Don't know**	**Unimportant**	**Should not be included**
204	Discuss with the suicidal person what actions they should take to get help.					
205	Encourage the suicidal person to get appropriate professional help as soon as possible (i.e. see a mental health professional or someone at a mental health service).					
206	Seek the permission of the suicidal person to contact their regular doctor or mental health professional about their concerns.					

207	Call a mental health centre or crisis telephone line and ask for advice on the situation.					
208	Call their doctor/GP, psychiatrist or other professional right away for the suicidal person.					
209	Get the suicidal person to phone an emergency number (i.e. Emergency services, a suicide helpline/mental health helpline, emergency mental health services).					
210	Phone an emergency number without letting the suicidal person know.					
211	Take the suicidal person to the nearest safe place (e.g. hospital emergency department).					
	**If the suicidal person has a specific plan, the first aider should:**	**Essential**	**Important**	**Don't know**	**Unimportant**	**Should not be included**
212	Discuss with the suicidal person what actions they should take to get help.					
213	Encourage the suicidal person to get appropriate professional help as soon as possible (i.e. see a mental health professional or someone at a mental health service).					
214	Seek the permission of the suicidal person to contact their regular doctor or mental health professional about their concerns.					
215	Call a mental health centre or crisis telephone line and ask for advice on the situation.					
216	Call their doctor/GP, psychiatrist or other professional right away for the suicidal person.					

217	Get the suicidal person to phone an emergency number (i.e. Emergency services, a suicide helpline/mental health helpline, emergency mental health services).					
218	Phone an emergency number without letting the suicidal person know.					
219	Take the suicidal person to the nearest safe place (e.g. hospital emergency department).					
	**If the suicidal person has the means to carry out their suicide plan/If the suicidal person does not agree to give the first aider the things they intend to use to kill themselves, the first aider should:**	**Essential**	**Important**	**Don't know**	**Unimportant**	**Should not be included**
220	Discuss with the suicidal person what actions they should take to get help.					
221	Encourage the suicidal person to get appropriate professional help as soon as possible (i.e. see a mental health professional or someone at a mental health service).					
222	Seek the permission of the suicidal person to contact their regular doctor or mental health professional about their concerns.					
223	Call a mental health centre or crisis telephone line and ask for advice on the situation.					
224	Call their doctor/GP, psychiatrist or other professional right away for the suicidal person.					
225	Get the suicidal person to phone an emergency number (i.e. Emergency services, a suicide helpline/mental health helpline, emergency mental health services).					

226	Phone an emergency number without letting the suicidal person know.					
227	Take the suicidal person to the nearest safe place (e.g. hospital emergency department).					
228	Ask the suicidal person if they would like the first aider to contact someone for them, such as a friend, family member, or trusted religious leader (such as Imams).					
	**If the suicidal person has attempted suicide in the past, the first aider should:**	**Essential**	**Important**	**Don't know**	**Unimportant**	**Should not be included**
229	Discuss with the suicidal person what actions they should take to get help.					
230	Encourage the suicidal person to get appropriate professional help as soon as possible (i.e. see a mental health professional or someone at a mental health service).					
231	Seek the permission of the suicidal person to contact their regular doctor or mental health professional about their concerns.					
232	Call a mental health centre or crisis telephone line and ask for advice on the situation.					
233	Call their doctor/GP, psychiatrist or other professional right away for the suicidal person.					
234	Get the suicidal person to phone an emergency number (i.e. Emergency services, a suicide helpline/mental health helpline, emergency mental health services).					
235	Phone an emergency number without letting the suicidal person know.					

236	Take the suicidal person to the nearest safe place (e.g. hospital emergency department).					
	**If the suicidal person is known to have a diagnosis of a mental illness (particularly of a psychotic disorder), the first aider should:**	**Essential**	**Important**	**Don't know**	**Unimportant**	**Should not be included**
237	Discuss with the suicidal person what actions they should take to get help.					
238	Encourage the suicidal person to get appropriate professional help as soon as possible (i.e. see a mental health professional or someone at a mental health service).					
239	Seek the permission of the suicidal person to contact their regular doctor or mental health professional about their concerns.					
240	Call a mental health centre or crisis telephone line and ask for advice on the situation.					
241	Call their doctor/GP, psychiatrist or other professional right away for the suicidal person.					
242	Get the suicidal person to phone an emergency number (i.e. Emergency services, a suicide helpline/mental health helpline, emergency mental health services).					
243	Phone an emergency number without letting the suicidal person know.					
244	Take the suicidal person to the nearest safe place (e.g. hospital emergency department).					
	**If the suicidal person refuses professional help, the first aider should :**	**Essential**	**Important**	**Don't know**	**Unimportant**	**Should not be included**

245	Discuss with the suicidal person what actions they should take to get help.					
246	Encourage the suicidal person to get appropriate professional help as soon as possible (i.e. see a mental health professional or someone at a mental health service).					
247	Seek the permission of the suicidal person to contact their regular doctor or mental health professional about their concerns.					
248	Call a mental health centre or crisis telephone line and ask for advice on the situation.					
249	Call their doctor/GP, psychiatrist or other professional right away for the suicidal person.					
250	Get the suicidal person to phone an emergency number (i.e. Emergency services, a suicide helpline/mental health helpline, emergency mental health services).					
251	Phone an emergency number without letting the suicidal person know.					
252	Take the suicidal person to the nearest safe place (e.g. hospital emergency department).					
253	If the first aider needs to contact a health professional about the suicidal person, they should preferably contact a professional the person already knows and trusts.					
254	If the first aider has to call the police, they should inform them that the person is suicidal in order to help them respond appropriately.					

255	As suicide is illegal in Pakistan, the police should not be involved except where their intervention is necessary (e.g. the person is about to jump from a building).					
	**If the suicidal person is reluctant to get professional help, the first aider should:**	**Essential**	**Important**	**Don't know**	**Unimportant**	**Should not be included**
256	Convince the person that they need help during crisis.					
257	Convince the person that they will not be judged or blamed for seeing a mental health professional.					
258	Encourage the suicidal person to get appropriate professional help as soon as possible (i.e. see a mental health professional or someone at a mental health service).					
259	Identify their significant others (family or friends).					
260	Contact their significant others (family or friends) to accompany them.					
261	Call a mental health center, or a crisis telephone helpline, and ask for advice on the situation, without letting the suicidal person know if needed.					
262	Call their doctor/GP, psychiatrist or other professional right away for the suicidal person and take the suicidal person to a hospital emergency department if needed.					
263	Get the suicidal person to phone an emergency number (i.e. Emergency services, a suicide helpline/mental health helpline, emergency mental health services).					

264	If the first aider needs to contact a health professional about the suicidal person, they should preferably contact a professional the person already knows and trusts.					
265	If the first aider has to call the police, they should inform them that the person is suicidal in order to help them respond appropriately.					
266	Seek an agreement with them that they will contact a specific person within a specified timeframe.					
267	Contact a suicide prevention hotline for guidance on how to help them.					
268	Talk to a health professional for advice on the situation.					
269	Contact emergency services on their behalf.					
270	Make sure someone who is close to the suicidal person is aware of the situation (i.e. close friend or family member).					
271	If the suicidal person doesn't want to talk to someone face-to-face, the first aider should encourage them to contact a suicide helpline.					
272	The first aider should find out information on the resources and services available for a person who is considering suicide.					
273	If the suicidal person is willing to seek professional help for their suicidal thoughts, the first aider should help them plan what they will say.					
	**If the suicidal person has a weapon, the first aider should:**	**Essential**	**Important**	**Don't know**	**Unimportant**	**Should not be included**
274	Try to take it away from them.					

275	Ask them to hand over the weapon.					
276	Remove themselves from the situation.					
277	Contact the police and inform them that the person is suicidal in order to help them respond appropriately.					
278	Seek help from family members, neighbors or others to remove it.					
279	Try to take it away from them while paying attention to their own safety.					
280	If possible, the first aider should measure out prescription medication so that the suicidal person only has a certain amount available (e.g. couple of days' worth).					
281	Encourage them to keep the weapon/dangerous objects out of reach should the talk of suicide occur over the phone.					
282	Contact emergency number so that the person is not alone when the talk of suicide occurs over the phone.					
283	The first aider must keep in mind that they may not be successful in preventing suicide.					
284	Not try to take it away from them.					
285	Please use the space below to suggest at least three first aid actions that you feel are important for providing initial assistance to suicidal people in Pakistan (e.g. cultural, social and religious issues).					
285.1						
285.2						

285.3						
286	Do you have any comments on these statements? Is there anything you would like to add to this section? Please write your ideas in the box provided.					
	**END OF SECTION 3**					
	**SECTION 4. TALKING TO A SUICIDAL PERSON**					
	This section contains statements about talking to the suicidal person in Pakistan about their suicidal thoughts. Please rate how important (from essential to should not be included) you think it is that each statement be included in the final guidelines.					
	**Letting them know you care - The first aider should:**	**Essential**	**Important**	**Don't know**	**Unimportant**	**Should not be included**
287	Tell the suicidal person they care and want to help.					
288	Tell the suicidal person that they do not want them to die or that they don’t want to lose them.					
289	Remind the suicidal person that they are loved and would be missed.					
290	Remind the suicidal person that they are worthy and their life is worthy.					
291	Reassure the suicidal person that they want to hear whatever the person has to say.					
292	Give the suicidal person their undivided attention.					

293	Be patient and calm while the suicidal person is talking about their feelings.					
294	Be patient and give the suicidal person time to get to the topic about their suicidal thoughts.					
295	Be conscious of their body language, ensuring it doesn't communicate a lack of interest or negative attitude.					
296	Keep in mind that asking too many questions can provoke anxiety in the suicidal person.					
297	Show they are listening by summarizing what the suicidal person is saying.					
298	Clarify important points with the person to make sure they fully understand.					
299	Ask what the suicidal person is thinking and feeling.					
300	Be supportive and understanding of the suicidal person.					
301	Express empathy for the suicidal person (e.g. "I understand how you feel").					
302	Listen to the suicidal person without expressing judgment.					
303	Avoid telling the person just to "be patient" as they might feel not-understood.					
	**Actively listening - The first aider should:**	**Essential**	**Important**	**Don't know**	**Unimportant**	**Should not be included**
304	Encourage the suicidal person to do most of the talking.					
305	Let the suicidal person know that it's okay to talk about things that might be painful.					

306	Ask open questions to find out more about the suicidal thoughts and feelings and the problems behind these.					
307	Share their thoughts with the suicidal person without expressing judgement.					
308	Focus on the things that will keep the suicidal person safe for now rather than the things that put the person at risk.					
309	Avoid discussion of any mental health problems experienced by the suicidal person, focusing instead on the reasons behind the suicide crisis.					
	**Reacting to suicidal thoughts**	**Essential**	**Important**	**Don't know**	**Unimportant**	**Should not be included**
310	Suicidal thoughts are often a plea for help and a desperate attempt to escape from problems and distressing feelings.					
311	The first aider should therefore allow the suicidal person to talk about those thoughts and feelings.					
312	The first aider needs to allow the suicidal person to talk about their reasons for wanting to die.					
313	The first aider should not try to just distract the suicidal person like saying “let’s go out”.					
	**The first aider should:**	**Essential**	**Important**	**Don't know**	**Unimportant**	**Should not be included**
314	Encourage the suicidal person to discuss their reasons for dying and their reasons for living.					
315	Validate that they are considering both options.					

316	Emphasize that living is an option for them.					
317	Ask about issues that affect the immediate safety of the person who is suicidal.					
318	Listen to what the suicidal person is saying without agreeing or disagreeing with their behavior or point of view.					
319	Validate the suicidal person's thoughts and feelings and acknowledge that these may be hard to talk about.					
320	Say whatever they feel they need to in order to help the suicidal person decide against suicide, including the use of guilt and threats, e.g. telling them they will go to hell or they will ruin the lives of others if they die by suicide, what will their parents/children do?					
	**Offering re-assurance - The first aider should:**	**Essential**	**Important**	**Don't know**	**Unimportant**	**Should not be included**
321	Reassure the suicidal person that it's okay to feel the way they do.					
322	Offer hope and re-assurance that their feelings are temporary, that help is available and things will get better.					
323	Assure the suicidal person that the feelings they are experiencing are probably caused by a mental illness that can be treated.					
324	Reassure the suicidal person that they understand how badly they feel.					
325	Reassure the suicidal person that they are there for them and want to help.					

326	Reassure the suicidal person that thoughts of suicide are common, that many people have them at some stage in their lives, and that it is possible to receive help.					
327	Reassure the suicidal person that it is not a crime, a sin or shame to feel suicidal.					
328	Reassure the suicidal person by letting them know that we all go through tough times.					
329	Reassure the suicidal person that the need for support and reaching out for help is the first step to feeling better.					
330	Remind the suicidal person that suicidal thoughts need not be acted on.					
331	Reassure the suicidal person that there are other alternatives to their problems rather than suicide.					
	**Highlighting protective factors**	**Essential**	**Important**	**Don't know**	**Unimportant**	**Should not be included**
332	The fact that the suicidal person is still alive, and talking to the first aider about their feelings, means that they are not quite sure about suicide. The first aider should point this out as a positive thing.					
333	If the first aider knows that the suicidal person has dreams or goals, remind them about those things. The first aider should encourage the suicidal person to stay alive so that they can make them come true.					
	**The first aider should:**	**Essential**	**Important**	**Don't know**	**Unimportant**	**Should not be included**

334	Thank the suicidal person for sharing their feelings with them and acknowledging the courage this takes.					
335	Discuss the 'good things' in a person's life, their hopes for the future, and other reasons to live.					
336	Find out what/who has supported the suicidal person in the past and whether these supports are still available.					
337	Encourage the suicidal person to think about their personal strengths and the positive things in their life.					
338	Remind the person of the good qualities they have.					
	**Helping with problem-solving**	**Essential**	**Important**	**Don't know**	**Unimportant**	**Should not be included**
339	The first aider should inquire about the problems the suicidal person is facing and how they can help.					
340	The first aider should indicate there are other alternatives aside from suicide.					
341	It is important that the first aider dispute the idea that suicide is the best or most viable solution.					
342	In order to reduce suicide risk, it is important for the first aider to try to solve the suicidal person's problems.					
343	By discussing specific problems, the first aider can help the person work out ways of dealing with the difficulties that seem insurmountable.					

344	The first aider should assist the suicidal person with problem-solving, by asking about the problem, identifying available resources, suggesting solutions and a plan of action.					
345	The first aider should help the suicidal person put their problems into perspective by reminding them that other people have much worse problems and still choose to live.					
346	The first aider should not try to provide a solution to the suicidal person's problems.					
	**Considering the consequences of suicide - The first aider should:**	**Essential**	**Important**	**Don't know**	**Unimportant**	**Should not be included**
347	Encourage the suicidal person to consider the consequences of suiciding, especially the effect it may have on the people they care about, for example, their family members.					
348	Remind the suicidal person that suicide is a permanent solution to a temporary problem.					
349	Accept the suicidal feelings for what they are and discuss suicide as a possibility rather than an unthinkable act.					
350	Make the suicidal person aware about the possible consequences the suicide attempt could have on their health if they survived it.					
351	Make the suicidal person aware of the negative stigma attached to suicide in society.					
	**Physical contact**	**Essential**	**Important**	**Don't know**	**Unimportant**	**Should not be included**

352	If the first aider has a close relationship with the suicidal person, they should show they care about them in a culturally appropriate manner by hugging them or holding their hand if both are of same gender. The first aider should keep in mind gender differences and cultural boundaries.					
353	The first aider should not touch (e.g. hug or hold hands with) the suicidal person unless they have a close personal relationship and/or of same gender.					
354	The first aider should not touch (e.g. hug or hold hands with) the suicidal person without their permission.					
	**Offering support**	**Essential**	**Important**	**Don't know**	**Unimportant**	**Should not be included**
355	The first aider should ask the suicidal person how they would like to be supported and if there is anything they can do to help.					
356	The first aider should not try to take on the suicidal person's responsibilities.					
357	The first aider should suggest things to distract the suicidal person from their suicidal thoughts, especially things which are relatively easy to do and which will encourage a sense of control and achievement.					
358	If the first aider is having trouble communicating with the suicidal person, they should ask simple questions, repeating these if necessary.					
	**Offer practical help/support**	**Essential**	**Important**	**Don't know**	**Unimportant**	**Should not be included**

359	The first aider should offer to help the suicidal person with positive practical tasks. This can give the person a chance to spend some time dealing with their situation or give them a chance for some rest.					
360	The first aider should offer to help the suicidal person to make plans or set goals for the future.					
361	The first aider should educate the suicidal persons' family members, friends, their religious/spiritual leader or significant other people about the suicide warning signs, risk and how they should assist the person.					
	**What to avoid when talking to the suicidal person**					
	**The first aider should not:**	**Essential**	**Important**	**Don't know**	**Unimportant**	**Should not be included**
362	Argue or debate with the person about their thoughts of suicide.					
363	Discuss with the person whether suicide is right or wrong.					
364	Minimize the suicidal person's problems.					
365	Give glib 'reassurance' such as 'don't worry', 'cheer up', 'you have everything going for you' or 'everything will be alright'.					
366	Interrupt with stories of their own.					
367	Call their bluff, dare or tell the suicidal person to 'just do it'.					
368	Attempt to give the suicidal person a diagnosis of a mental health problem.					

369	Use guilt or threats to prevent suicide (e.g. do not tell the person that suicide is a sin and they will go to hell or ruin other people's lives if they die by suicide).					
370	Let the fear of saying the wrong words or of not saying the perfect words keep them from encouraging the suicidal person to talk.					
371	Take any hurtful actions or words of the suicidal person personally.					
372	The first aider should avoid giving advice.					
373	The first aider should avoid asking the suicidal person why they are having suicidal thoughts.					
374	If the suicidal person is thinking about taking an overdose (for example with rat, wheat or medical pills), the first aider should tell the person that overdosing can lead to messy, painful and long-drawn-out consequences, such as slow poisoning.					
375	Please use the space below to suggest at least three first aid actions that you feel are important when talking to a suicidal person in Pakistan (e.g. cultural, social, gender, age and religious issues).					
375.1						
375.2						
375.3						
376	Do you have any comments on these statements? Is there anything you would like to add to this section? Please write your ideas in the box provided.					

	**END OF SECTION 4**					
	**Section 5. Safety planning with suicidal people**					
	The statements in this section are about the development of a plan to keep the suicidal person safe.<br>Please keep in mind that a Safety plan is an agreement between the suicidal person and the first aider that involves actions to keep the suicidal person safe.<br>Please rate how important (from essential to should not be included) you think it is that each statement be included in the final guidelines					
	**Developing a safety plan with the suicidal person**	**Essential**	**Important**	**Don't know**	**Unimportant**	**Should not be included**
377	The first aider should develop a safety plan with the suicidal person.					
378	The first aider should engage the suicidal person to the fullest extent possible in decisions about a safety plan.					
	**What the plan should include**					
	**The safety plan should:**	**Essential**	**Important**	**Don't know**	**Unimportant**	**Should not be included**
379	Be clear, outlining what will be done, who will be doing it, and when it will be carried out.					
380	Focus more on what the suicidal person should do rather than what they should not do.					
381	Include an agreement that the suicidal person does not attempt suicide.					
382	Include an agreement that the suicidal person does not use any alcohol or other drugs.					

383	Be kept somewhere accessible to the suicidal person.					
384	The first aider should not assume that a safety plan is adequate to keep the suicidal person safe.					
	**Who to contact**	**Essential**	**Important**	**Don't know**	**Unimportant**	**Should not be included**
385	The safety plan should include 24-hour safety contacts (such as the suicidal person's doctor or mental health care professional, a suicide helpline or crisis line, as well as friends and family members) who will help in an emergency.					
386	The first aider should ask the suicidal person to keep a list of safety contacts with them and agree to call someone when they are feeling suicidal.					
387	The first aider should work with the suicidal person to create plans to ensure their safety for the next 24, 48 and 72 hours.					
	**When to make a safety plan**	**Essential**	**Important**	**Don't know**	**Unimportant**	**Should not be included**
388	If the suicidal person won't make a safety plan, it is not safe to leave them alone for any period of time. The first aider should make sure someone stays close to the person (in the same room, in visual contact) and get outside help immediately.					
389	If the suicidal person won't make a safety plan, the first aider should get professional help immediately.					

390	The first aider shouldn't use a safety plan with a suicidal person they don't know well.					
391	The first aider should only make a safety plan with someone they know well.					
392	The first aider shouldn't use a safety plan with a suicidal person who is severely depressed.					
393	The first aider shouldn't use a safety plan with a suicidal person who is using drugs or alcohol.					
394	The first aider shouldn't use a safety plan with a suicidal person who is psychotic.					
395	Please use the space below to suggest at least three more first aid actions that you feel are important in safety planning with suicidal people (e.g. cultural, gender, social and religious/spiritual issues).					
395.1						
395.2						
395.3						
396	Do you have any comments on these statements? Is there anything you would like to add to this section? Please write your ideas in the box provided					
	**END OF SECTION 5**					
	**Section 6. Ensuring safety for suicidal people**					

	The statements in this section contain actions the first aider can take to help ensure the safety of the suicidal person.<br>Please rate how important (from essential to should not be included) you think it is that each statement be included in the final guidelines.					
		**Essential**	**Important**	**Don't know**	**Unimportant**	**Should not be included**
397	The first aider should make sure any potentially harmful items are not available to the suicidal person by removing access to these items.					
398	The first aider must gain the person's trust before removing the means of suicide.					
399	The first aider should try to remove the means of suicide available to the suicidal person if it is safe to do so.					
400	The first aider should ask the suicidal person to give them the things they intend using to kill themselves.					
401	If the suicidal person agrees to give the first aider the things they intend using to kill themselves, the first aider should dispose of them right away (i.e. flush pills and poison down the toilet, hand gun to the police, throw away razors or knives).					
402	If the suicidal person agrees to hand over the means of suicide, on the condition that they can have them back if they want them, the first aider should argue the point with them for as long as it takes.					

403	If the suicidal person agrees to hand over the means of suicide, on the condition that they can have them back if they want them, the first aider should agree to this.					
404	If the first aider can't get the suicidal person to agree to hand over the means of suicide (e.g. pills, poison such as wheat pills, rat pills, bleaching agents, gun, razor), emergency services must be contacted immediately.					
405	If the first aider can't get the suicidal person to agree to hand over the means of suicide (e.g. pills, gun, razor), they should try to take these things secretly.					
406	The first aider should help the suicidal person to decide who they can contact if they become suicidal again in the future.					
407	Please use the space below to suggest at least three first aid actions that you feel are important in ensuring safety among suicidal people (e.g. cultural, gender, social and religious/spiritual issues).					
407.1						
407.2						
407.3						
408	Do you have any comments on these statements? Is there anything you would like to add to this section? Please write your ideas in the box provided					

	**END OF SECTION 6**					
	**Section 7. Passing time during a crisis among suicidal people in Pakistan**					
	The statements in this section are about what the first aider should do with the suicidal person during the suicide crisis period. Please rate how important (from essential to should not be included) you think it is that each statement be included in the final guidelines.					
		**Essential**	**Important**	**Don't know**	**Unimportant**	**Should not be included**
409	Ask the suicidal person to postpone the decision to suicide.					
410	Develop a list with the suicidal person of other things they can do to distract themselves.					
411	Do something pleasant for the suicidal person. For example, cooking a favourite meal, watching a movie or listening to music with them.					
412	Encourage the suicidal person to undertake some relaxing activities, such as taking a hot bath, going for a long walk or reading something enjoyable.					
413	Encourage the suicidal person to do something active like going for a walk or a jog.					
414	Offer to join the person in some activity they normally enjoy.					

415	Encourage the suicidal person to take some sleeping pills, as they should be feeling better by the time they wake up.					
416	Encourage the suicidal person to spend time with their significant others (e.g. family, friends, or religious leaders).					
417	The first aider should not take the suicidal person to parties (or a get together) or places where people are having fun, as this could make them more depressed.					
418	During the suicidal crisis, the suicidal person and the first aider should be actively working on practical strategies to solve the life problems.					
419	The first aider and the suicidal person should find something to do together until the crisis has passed.					
420	It is preferable that the suicidal person choose an activity which has been found in the past to help them cope or that they enjoy.					
421	If appropriate or necessary, the first aider should involve trusted significant others in activities with the suicidal person to pass time.					
422	The first aider should ask if the suicial person is involved in spiritual and/or religious practices (e.g. pray, Wird/Zikr, Ijtema, etc) and encourage these practices to pass time.					
423	If the suicidal person wants to be left alone, and can assure the first aider of their safety, the first aider should agree.					

424	Please use the space below to suggest at least three first aid actions that you feel are important for passing time during a suicide crisis among suicidal people (e.g. cultural, gender, social and religious/spiritual issues).					
424.1						
424.2						
424.3						
425	Do you have any comments on these statements? Is there anything you would like to add to this section? Please write your ideas in the box provided					
	**END OF SECTION 7**					
	**Section 8.What the first aider should know in providing suicide first aid to a suicidal person in Pakistan**					
		**Essential**	**Important**	**Don't know**	**Unimportant**	**Should not be included**
426	Of how commonly suicide occurs.					
427	That there are many more suicide attempts than completed suicides.					
428	Of the risk factors for suicide.					
429	Of the link between suicide and mental illness.					
430	That talking about suicide will not 'put the idea' into someone's head.					

431	Of the reasons why people have thoughts about suicide.					
432	That most suicidal people do not want to die. They simply do not want to live with the pain.					
433	That suicidal people believe they have no choice but to die by suicide.					
434	That anyone could have thoughts of suicide.					
435	That suicidal thoughts are temporary.					
436	That suicidal behaviour is a cry for help.					
437	Of the reasons why people who are having suicidal thoughts don't ask for help.					
438	That people thinking about suicide are not likely to seek help, but do show warning signs to their family and friends.					
439	That suicide can be prevented.					
440	That openly talking about suicidal thoughts and feelings can save a life.					
441	That they should not underestimate their abilities to help a suicidal person, even to save a life.					
442	That unless someone tells you, the only way to know if a person is thinking of suicide is to ask.					
443	That use of alcohol or other drugs can increase the risk of a person acting on suicidal thoughts.					
444	That even though the first aider can offer support, they are not responsible for the actions or behaviour of someone else, and cannot control what they might decide to do.					

445	Of the local services that can assist in response to people at risk of suicide (i.e. hospitals, mental health clinics, mobile outreach crisis teams, suicide prevention helplines, local emergency services).					
446	Please use the space below to suggest at least three first aid actions that you feel are important for what the first aider should know when providing suicide first aid to suicidal people in Pakistan (e.g. cultural, gender, social and religious/spiritual issues).					
446.1						
446.2						
446.3						
447	Do you have any comments on these statements? Is there anything you would like to add to this section? Please write your ideas in the box provided					
	**END OF SECTION 8**					
	**Section 9: Confidentiality among suicidal people in Pakistan**<br>The statements in this section are about the confidentiality of the thoughts and behaviours of a suicidal person in Pakistan. Please rate how important (from essential to should not be included) you think it is that each statement be included in the final guidelines					
	.	**Essential**	**Important**	**Don't know**	**Unimportant**	**Should not be included**

448	The first aider should not keep the person's suicidal thoughts a secret from potential helpers, but should discuss with the person which specific information should be confidential.					
449	The first aider must never agree to keep the suicidal person's suicidal plans a secret.					
450	The first aider should tell the immediate family/close ones about the person's intention to suicide.					
451	If the suicidal person doesn't want the first aider to tell anyone about their suicidal thoughts, the first aider should not agree and explain why, e.g. "I care about you too much to keep a secret like this. You need help and I am here to help you get it".					
452	If the suicidal person asks the first aider to keep the discussion about suicide a secret, the first aider should keep the secret.					
453	If the suicidal person asks the first aider to promise they will keep the discussion about suicide a secret, they should agree, but tell someone else anyway.					
454	The first aider should not keep the person's suicidal thoughts a secret from potential helpers, but should discuss with the person whether other details should be confidential.					
455	The first aider should treat everything the suicidal person says in complete confidence.					

456	The first aider should treat the suicidal person with respect and involve them in decisions about who else knows about the suicidal crisis.					
457	The first aider should try to convince the suicidal person that it is better to not keep their suicidal intentions a secret but involve someone else (e.g. a professional, a trustworthy friend or a family member, teacher, religious leader).					
458	If the suicidal person refuses to give permission to disclose information about their suicidal thoughts, the first aider may need to breach their confidentiality to ensure their safety.					
459	The first aider should keep in mind that it is much better to have the person angry at them for sharing their suicidal thoughts without their permission in order to obtain help than to lose the person to suicide.					
460	The first aider should ask for help from the person’s relatives, friends or house mates to ensure the person does not have access to weapons, poisons, or other means for suicide.					
461	If the suicidal person is a minor, the first aider must make their guardians (i.e. the family or the social welfare) aware of the person’s intentions to kill themselves.					

462	If the first aider decides to involve a professional or someone else, they should inform the suicidal person of their decision and explain that this is necessary to ensure their safety.					
463	Please use the space below to suggest at least three first aid actions that you feel are important to confidentiality among people in Pakistan (e.g. cultural, social, religious, gender, age related issues).					
463.1						
463.2						
463.3						
464	Do you have any comments on these statements? Is there anything you would like to add to this section? Please write your ideas in the box provided					
	**END OF SECTION 9**					
	**SECTION 10. SPECIFIC TO ADOLESCENTS**					
	Because the support provided to young people might be quite different to the support provided to an adult, the statements in this section are to be rated for providing suicide first aid to an adolescent in Pakistan. The term adolescent refers to a young person aged between 12 and 18 years (nominally the high school years). Please rate how important (from essential to should not be included) you think it is that each statement be included in the final guidelines.					

	Assessing seriousness of suicide risk in adolescents in Pakistan	Essential	Important	Don't know	Unimportant	Should not be included
465	If the suicidal adolescent says that the situation is not serious or that they can handle it on their own, the first aider should respect this.					
	**Initial assistance to adolescents in Pakistan**	**Essential**	**Important**	**Don't know**	**Unimportant**	**Should not be included**
466	The first aider should not leave an adolescent who is feeling suicidal on their own.					
467	The first aider does not need to be with the suicidal adolescent all the time, but should check on them regularly.					
	**If the adolescent is suicidal, the first aider should:**	**Essential**	**Important**	**Don't know**	**Unimportant**	**Should not be included**
468	Discuss with the suicidal person what actions they should take to get help.					
469	Encourage the suicidal person to get appropriate professional help as soon as possible (i.e. see a mental health professional or someone at a mental health service, talk to a school counselor).					
470	Seek the permission of the suicidal person to contact their regular doctor or mental health professional about their concerns.					
471	Call a mental health centre or crisis telephone line and ask for advice on the situation.					
472	Call their doctor/GP, psychiatrist or other professional right away for the suicidal person.					

473	Ask the suicidal adolescent if they would like the first aider to contact someone they are close to for them such as a trusted friend or a family member.					
474	Get the suicidal person to phone an emergency number (i.e. Emergency services, a suicide helpline, emergency mental health services).					
475	Phone an emergency number without letting the suicidal person know.					
476	Take the suicidal person to a hospital emergency department.					
477	Discuss with the suicidal person what actions they should take to get help.					
478	If the suicidal person is a minor, the first aider must make their significant elders aware of the person's intention to kill themselves.					
	**If the suicidal adolescent can't commit to stay safe, the first aider should:**	**Essential**	**Important**	**Don't know**	**Unimportant**	**Should not be included**
479	Discuss with the suicidal person what actions they should take to get help.					
480	Encourage the suicidal person to get appropriate professional help as soon as possible (i.e. see a mental health professional or someone at a mental health service, school counsellor).					
481	Seek the permission of the suicidal person to contact their regular doctor or mental health professional about their concerns.					
482	Call a mental health centre or crisis telephone line and ask for advice on the situation.					

483	Call their doctor/GP, psychiatrist or other professional right away for the suicidal person.					
484	Ask the suicidal adolescent if they would like the first aider to contact someone they are close to for them such as a trusted friend or a family member.					
485	Get the suicidal person to phone an emergency number (i.e. Emergency services, a suicide					
486	Phone an emergency number without letting the suicidal person know.					
487	Take the suicidal person to a hospital emergency department.					
488	The first aider should treat the suicidal adolescent with respect and involve them in decisions about who else knows about the suicidal crisis.					
	**If the suicidal adolescent has a specific plan, the first aider should:**	**Essential**	**Important**	**Don't know**	**Unimportant**	**Should not be included**
489	Discuss with the suicidal person what actions they should take to get help.					
490	Encourage the suicidal person to get appropriate professional help as soon as possible (i.e. see a mental health professional or someone at a mental health service).					
491	Seek the permission of the suicidal person to contact their regular doctor or mental health professional about their concerns.					
492	Call a mental health centre or crisis telephone line and ask for advice on the situation.					

493	Call their doctor/GP, psychiatrist or other professional right away for the suicidal person.					
494	Get the suicidal person to phone an emergency number (i.e. Emergency services, a suicide helpline, emergency mental health services).					
495	Phone an emergency number without letting the suicidal person know.					
496	Take the suicidal person to a hospital emergency department.					
	**If the suicidal adolescent has the means to carry out their suicide plan , the first aider should:**	**Essential**	**Important**	**Don't know**	**Unimportant**	**Should not be included**
497	Discuss with the suicidal person what actions they should take to get help.					
498	Encourage the suicidal person to get appropriate professional help as soon as possible (i.e. see a mental health professional or someone at a mental health service, a school counselor).					
499	Seek the permission of the suicidal person to contact their regular doctor or mental health professional about their concerns.					
500	Call a mental health centre or crisis telephone line and ask for advice.					
501	Call their doctor/GP, psychiatrist or other professional right away for the suicidal person.					

502	Get the suicidal person to phone an emergency number (i.e. Emergency services, a suicide helpline, emergency mental health services etc).					
503	Phone an emergency number without letting the suicidal person know.					
504	Take the suicidal person to a hospital emergency department.					
	**If the suicidal adolescent does not agree to give the first aider the things they intend using to kill themselves, the first aider should:**	**Essential**	**Important**	**Don't know**	**Unimportant**	**Should not be included**
505	Discuss with the suicidal person what actions they should take to get help.					
506	Encourage the suicidal person to get appropriate professional help as soon as possible (i.e. see a mental health professional or someone at a mental health service, a school counselor).					
507	Seek the permission of the suicidal person to contact their regular doctor or mental health professional about their concerns.					
508	Call a mental health centre or crisis telephone line and ask for advice.					
509	Call their doctor/GP, psychiatrist or other professional right away for the suicidal person.					
510	Get the suicidal person to phone an emergency number (i.e. Emergency services, a suicide helpline, emergency mental health services etc).					

511	Phone an emergency number without letting the suicidal person know.					
512	Take the suicidal person to a hospital emergency department.					
	**If the suicidal adolescent has attempted suicide in the past, the first aider should:**	**Essential**	**Important**	**Don't know**	**Unimportant**	**Should not be included**
513	Discuss with the suicidal person what actions they should take to get help.					
514	Encourage the suicidal person to get appropriate professional help as soon as possible (i.e. see a mental health professional or someone at a mental health service, a school counselor).					
515	Seek the permission of the suicidal person to contact their regular doctor or mental health professional about their concerns.					
516	Call a mental health centre or crisis telephone line and ask for advice.					
517	Call their doctor/GP, psychiatrist or other professional right away for the suicidal person.					
518	Get the suicidal person to phone an emergency number (i.e. Emergency services, a suicide helpline, emergency mental health services etc).					
519	Phone an emergency number without letting the suicidal person know.					
520	Take the suicidal person to a hospital emergency department.					

	If the suicidal adolescent is known to have a diagnosis of a mental illness (particulalrly a psychotic diagnosis), the first aider should:	Essential	Important	Don't know	Unimportant	Should not be included
521	Discuss with the suicidal person what actions they should take to get help.					
522	Encourage the suicidal person to get appropriate professional help as soon as possible (i.e. see a mental health professional or someone at a mental health service, a school counselor).					
523	Seek the permission of the suicidal person to contact their regular doctor or mental health professional about their concerns.					
524	Call a mental health centre or crisis telephone line and ask for advice.					
525	Call their doctor/GP, psychiatrist or other professional right away for the suicidal person.					
526	Get the suicidal person to phone an emergency number (i.e. Emergency services, a suicide helpline, emergency mental health services etc).					
527	Phone an emergency number without letting the suicidal person know.					
528	Take the suicidal person to a hospital emergency department.					
	**If the suicidal adolescent refuses professional help, the first aider should:**	**Essential**	**Important**	**Don't know**	**Unimportant**	**Should not be included**
529	Discuss with the suicidal person what actions they should take to get help.					

530	Encourage the suicidal person to get appropriate professional help as soon as possible (i.e. see a mental health professional or someone at a mental health service, a school counselor).					
531	Seek the permission of the suicidal person to contact their regular doctor or mental health professional about their concerns.					
532	Call a mental health centre or crisis telephone line and ask for advice.					
533	Call their doctor/GP, psychiatrist or other professional right away for the suicidal person.					
534	Get the suicidal person to phone an emergency number (i.e. Emergency services, a suicide helpline, emergency mental health services etc).					
535	Phone an emergency number without letting the suicidal person know.					
536	Take the suicidal person to a hospital emergency department.					
	**If the suicidal adolescent is reluctant to seek help, the first aider should:**	**Essential**	**Important**	**Don't know**	**Unimportant**	**Should not be included**
537	Keep encouraging them to see a mental health professional.					
538	Seek an agreement with them that they will contact a specific person within a specified timeframe.					
539	Contact a suicide prevention hotline for guidance on how to help them.					

540	Talk to a health professional for advice on the situation.					
541	Contact emergency services on their behalf.					
542	Make sure someone who is close to the suicidal adolescent is aware of the situation (i.e. close friend or family member).					
543	If the first aider is unable to persuade the suicidal adolescent to get help, they should get assistance from a trusted friend, helpline or mental health professional.					
544	The first aider should ensure that the suicidal adolescent receives help from a health professional, support group or relevant community organization.					
	**Talking with a suicidal adolescent in Pakistan**	**Essential**	**Important**	**Don't know**	**Unimportant**	**Should not be included**
545	The first aider should avoid giving advice to the suicidal adolescent.					
546	In order to reduce suicide risk, it is important for the first aider to try to solve the suicidal adolescent's problems.					
547	The first aider should not try to take on the suicidal adolescent's responsibilities.					
	**Safety planning with adolescents in Pakistan**	**Essential**	**Important**	**Don't know**	**Unimportant**	**Should not be included**
548	The first aider should develop a safety plan with the suicidal adolescent.					

549	If the suicidal adolescent won't make a safety plan, it is not safe to leave them alone for any period of time. The first aider should make sure someone stays close by to the person (in the same room, in visual contact) and get whatever outside resources are available (e.g. family, emergency mental health care if necessary).					
550	If the suicidal adolescent won't make a safety plan, the first aider should get professional help immediately.					
	**Passing time during the crisis among adolescents in Pakistan**	**Essential**	**Important**	**Don't know**	**Unimportant**	**Should not be included**
551	If the suicidal adolescent wants to be left alone, and can assure the first aider of their safety, the first aider should agree.					
	**Confidentiality among adolescents in Pakistan**	**Essential**	**Important**	**Don't know**	**Unimportant**	**Should not be included**
552	The first aider should treat the suicidal adolescent with respect and involve them in decisions about who else knows about the suicidal crisis.					
553	If the suicidal adolescent asks the first aider to promise they will keep the discussion about suicide a secret, they should agree, but tell someone else anyway.					
554	Please use the space below to suggest at least three first aid actions that you feel are important in providing mental health first aid to adolescents in Pakistan (e.g. cultural, social and religious issues).					

554.1						
554.2						
554.3						
555	Do you have any comments on these statements? Is there anything you would like to add to this section? Please write your ideas in the box provided					
	**END OF SECTION 10**					
	**SECTION 11. GENDER SPECIFIC**					
	The following section contains gender-specific statements about what the first aider should do with the suicidal adolescent during the crisis. Please rate each statement according to how important you believe it is as a potential Mental Health First Aid guideline for helping a suicidal person in Pakistan.					
	**Female**	**Essential**	**Important**	**Don't know**	**Unimportant**	**Should not be included**
556	The first aider should be aware of different risk factors for a suicidal woman such as domestic violence, postnatal depression, and interpersonal conflicts.					

557	The first aider should be aware that females are more likely to discuss physical complaints with no apparent physical source when in fact they are having suicidal thoughts.					
558	The first aider should be aware that with females, it is important to discuss relationships issues, sexual interactions and related concerns in a sensitive manner.					
559	The first aider should be aware that females from some cultural backgrounds may not be in a position to seek professional help and therefore family members must be involved.					
560	If the first aider thinks the person is uncomfortable interacting with them due to difference in gender, they should ask the person if they would prefer to talk to someone of the same gender.					
	**Male**	**Essential**	**Important**	**Don't know**	**Unimportant**	**Should not be included**
561	The first aider should be aware of different risk factors among a male suicidal person such as alcohol misuse, substance abuse, and financial difficulties.					
562	The first aider should be aware that some males may be less likely to express their emotions and open up about suicidal intentions.					

563	The first aider must be aware that Pakistani men may not openly disclose previous suicide attempts and may instead state, for example, that they had an "accidental overdose of medication or poison".					
564	The first aider should be aware that increased expression of emotions in males, such as crying or aggressive behaviors, could indicate suicide risk.					
565	Please use the space below to suggest at least three gender specific statements that you feel are important for assisting a suicidal person in Pakistan.					
565.1						
565.2						
565.3						
566	Do you have any comments on these statements? Is there anything you would like to add to this section? Please write your ideas in the box provided					
